# Trends of public health research output from India during 2001-2008

**DOI:** 10.1186/1741-7015-7-59

**Published:** 2009-10-14

**Authors:** Lalit Dandona, Magdalena Z Raban, Rama K Guggilla, Aarushi Bhatnagar, Rakhi Dandona

**Affiliations:** 1 Public Health Foundation of India, New Delhi, India; 2 Institute for Health Metrics and Evaluation, University of Washington, Seattle, Washington, USA; 3 School of Public Health and George Institute for International Health, University of Sydney, Sydney, New South Wales, Australia; 4 George Institute for International Health - India, Hyderabad, India

## Abstract

**Background:**

An understanding of how public health research output from India is changing in relation to the disease burden and public health priorities is required in order to inform relevant research development. We therefore studied the trends in the public health research output from India during 2001-2008 that was readily available in the public domain.

**Methods:**

The scope and type of the published research from India in 2007 that was included in the PubMed database was assessed and compared with a previous similar assessment for 2002. Papers were classified based on the review of abstracts and original public health research papers were assessed in detail. Impact factors for the journals were used to compute quality-adjusted research output. The websites of governmental organizations, academic and research institutions and international organizations were searched in order to identify and review reports on original public health research produced in India from 2001 to 2008. The reports were classified based on the topics covered and quality and their trends over time were assessed.

**Results:**

The number of original health research papers from India in PubMed doubled from 4494 in 2002 to 9066 in 2007. This included a 3.1-fold increase in public health research papers, but these comprised only 5% of the total papers in 2007. Within public health, the increase was lowest for the health system and policy category. Several major causes of disease burden in India continued to be underrepresented in the quality-adjusted public health research output in 2007. The number of papers evaluating population health interventions increased from 2002 to 2007, but there were none on the leading non-communicable causes of disease burden or on road traffic injuries. The number of identified original public health research reports increased by 64.7% from 204 in 2001-2004 to 336 in 2005-2008. The proportion of reports on reproductive and child health was very high but decreased slightly from 38.7% of the total in 2001-2004 to 31.5% in 2005-2008 (*P *= 0.09); those on the leading chronic non-communicable conditions and injuries increased from 6.4% to 13.4% (*P *= 0.01) but this was still much lower than their contribution to the disease burden. Health system/policy issues were the topic in 27.4% reports but health information issues were covered in a miniscule 0.6% reports. The proportion of reports that were evaluations increased slightly from 26% in 2001-2004 to 31.5% in 2005-2008, with this proportion being higher among the reports commissioned by international organizations (*P *< 0.001). The proportion of reports commissioned by Indian governmental organizations alone, or in collaboration with international organizations, doubled from 2001-2004 to 2005-2008 (*P *< 0.001). Only 25% of the total 540 reports had a quality score of adequate or better. The quality of reports produced by collaborations between Indian and international organizations was higher than those produced by Indian or international organizations alone (*P *< 0.001).

**Conclusion:**

This is the first analysis from India that includes research reports in addition to published papers. It provides the most up-to-date understanding of public health research output from India. The increase in available public health research output and the increase in commissioning of this research by Indian governmental organizations are encouraging. However, the distribution of research topics and the quality of research reports continue to be unsatisfactory. It is necessary for health policy to address these continuing deficits in public health research in order to reduce the very large disease burden in India.

## Background

Our previously reported analysis of published research output from India in 2002 highlighted the inadequacies of the output at that time [1]. This analysis was based on papers published from India that were included in the PubMed bibliographic database. Of the original health research papers from India in 2002 in PubMed, only 3.3% were on public health. Within this low proportion, several major causes of disease burden across all three categories - communicable and non-communicable diseases and injuries - were grossly under-representation compared to their contribution to the disease burden as a large proportion of the research output did not relate to the major causes of the disease burden [1,2]. In addition, human resources, health policy and impact evaluations of interventions were particularly poorly represented [1]. These deficiencies and mismatches in published research output from India were subsequently discussed [3-5].

While basic science, clinical health and public health research all contribute to the necessary evidence base for improving the health of societies, public health research enables understanding of the distribution of health and disease in the population, the determinants of this distribution and the ways in which health system and policies could reduce the disease burden. This important component necessary for informed planning of how to improve population health has been weak in India, leading to gaps in health system and policy development [1,6,7]. In this paper we analyse the published research output from India in 2007, using methods similar to those that we used for 2002, in order to examine if there have been any significant changes in the major deficiencies that were identified five years ago. In addition, we report the findings derived from a review of reports on public health research produced in India from 2001 to 2008 that were commissioned or initiated by governmental, international or other funding organizations, or by academic institutions, and which were available in the public domain on the internet. Together, these analyses provide the most up-to-date understanding of the public health research output from India.

## Methods

Health research was defined as research related to human health. Published papers on health research produced from India were accessed from a bibliographic database and public health research reports from India were accessed from the websites of various organizations, as described below.

### Research published in journals

PubMed, the database of the US National Library of Medicine [8], is one of the most widely used online health literature bibliographic database in the world. We utilized this to access the published research output from India in 2007 using methods similar to those used for an earlier assessment from India in 2002 [1]. We searched PubMed for papers published from India in 2007 using 'India' in the author affiliation option. As PubMed gives the institutional affiliation and its location only for the first author, papers that had the first author affiliated with an Indian institution were considered as research output from India. Only papers with abstracts were included in this assessment. A paper was classified as 'original research' if it included an original analysis of primary or secondary data. Only original research papers were analysed. Abstracts were reviewed in order to classify the papers in various categories of the type of research, the disease/condition covered and the type of institution to which the first author was affiliated.

Research papers were classified as basic health research, clinical health research or public health research and put into further sub-categories based on the following criteria. Basic health research was classified as 'pure' if it dealt with experimental or theoretical work to advance health knowledge without a defined specific application or 'applied' if it did have such an application. Clinical health research was classified as: 'patient series/management' if the paper was about clinical cases or issues on the management of patients; 'laboratory study/clinical investigation' if it dealt mainly with laboratory analysis of patient specimens or only clinical investigations of patients; 'clinical trial' if it was a trial of a clinical intervention in a health facility setting; and 'clinical epidemiology' if it was about the distribution or determinants of disease assessed in a health facility setting. Public health research papers were reviewed in full for a more detailed assessment, and classified as 'epidemiology', 'behavioural/environmental/social' or 'health system/policy'. Epidemiology included papers that dealt with the study of the distribution or determinants of disease or health in the population or methodological issues in epidemiological assessments. Behavioural/environmental/social research included papers that dealt with an understanding of health behaviour or its modification to promote health, environmental influences on health, occupational health safety or the social dimensions of health. Health system/policy research included papers that addressed health service provision, human resources for health, monitoring or surveillance of disease or health, health information system, health economics or finance, or policy/governance to improve population health. A public health research paper was considered to be an 'evaluation' if it dealt with assessment of the process, outcome or impact of an intervention, programme or policy to improve population health.

Classification of each paper was attempted under the disease/condition that it covered, according to the classification used in the Global Burden of Disease Project [2]. If a paper covered generic issues which could not be classified under a particular disease/condition, it was considered unclassifiable for disease/condition. The 2007 impact factor of the journal in which each paper was published was used as a surrogate indicator of quality [9,10]. The percent quality-adjusted research output for papers in a disease/condition category was calculated as follows:



The proportion of the quality-adjusted output for the diseases/conditions was compared with the proportion of disease burden caused by them as estimated for 2007 and projected for 2015 by the Global Burden of Disease Project (unpublished data provided by the World Health Organization).

IndMED [11], an online database that includes several Indian biomedical journals, was also searched but the abstracts/papers for all the months of 2007 were not available in this database, which was what we had found for the 2002 assessment also. Therefore, this database could not be included in this study. Various aspects of the 2007 original health research output from India included in PubMed were compared with the output in 2002 [1].

### Reports on public health research

We searched reports on original public health research, as defined above, which were authored by India-based organizations and were available in the public domain on the internet. International organizations with a base in India were included. Our recent review of the essential health information available from India was utilized to enable us to begin the identification of the sources for these reports [12]. This included websites of ministries and other organizations of the government in India, academic institutions, international organizations and funding agencies involved in any form with public health. These initial website sources provided additional leads to other relevant organizations. The search engine Google was used to locate websites of the additional relevant organizations identified from the initial search of websites as potentially containing public health research reports. Each website was searched thoroughly by examining any available database of publications or reports and systematically reviewing all pages within the website for links to publications or reports. Over 200 websites were searched for original public health research reports produced from 2001 to 2008 (Additional file 1). For a few reports that did not mention the year of production, the year was assumed to be the one after the year of the latest reference cited in that report.

The identified public health research reports were reviewed and, based on the predominant thrust of their content, were classified under diseases/conditions corresponding to the national health programmes/themes, or health system components or other major population health themes [13,14]. A report was classified as an evaluation if it assessed the process, outcome or impact of an intervention, programme or policy to improve population health. If the report covered specific Indian states, whether it covered any of 16 underdeveloped states - Empowered Action Group states (Bihar, Chhattisgarh, Jharkhand, Madhya Pradesh, Orissa, Rajasthan, Uttarakhand, Uttar Pradesh) or north-east states (Arunachal Pradesh, Assam, Manipur, Meghalaya, Mizoram, Nagaland, Sikkim, Tripura) that together make up half of India's population [15] - was recorded.

The organizations that commissioned the research and the organizations that conducted them were classified as Indian or international. Indian organizations were further sub-categorized under government ministries and departments, academic and research institutions, not-for-profit organizations and international organizations under multilateral, bilateral and other.

Each report underwent a detailed quality assessment that included seven components: definition of objectives of the research; description of methods; appropriateness of methods; clarity of results; level of analysis; appropriateness of interpretation of the findings; and relevance of research to informing further improvements in public health. Based on the generally accepted quality norms for publications, each component was scored on a scale from 0 to 3, where 0 was completely inadequate, 1 was somewhat inadequate/not meeting reasonable standard, 2 was adequate/meeting reasonable standard, and 3 was excellent implying close to ideal. Before the actual scoring, many trial runs of this quality scoring were done by multiple investigators to arrive at consistency in this scoring. Considering the total maximum possible quality score of 21 (three for each of the seven components) as 100%, the total score for each report was converted into a percent score. Reports with a score of 33% or less were considered very inadequate, those with a score of 34-66% were considered somewhat inadequate and those with a score of 67% or more were considered adequate or of better quality.

Trends were assessed from 2001-2004 to 2005-2008 for: the topics covered by the reports; proportion of reports that were evaluations; the inclusion of under-developed states in the research; the commissioning organizations; the institutions that conducted the research; and the quality of reports. The relationship between some of these variables with one another was assessed. The chi-square test was used to assess statistical significance where appropriate.

Data were entered in MS Access and MS Excel databases and analysed using SPSS software (SPSS Inc, Chicago, USA).

## Results

### Research published in journals

Of all papers included in PubMed, 1.64% had a first author from India in 2007 compared with 1.11% in 2002 (*P *< 0.001). There were 9066 original health research papers in PubMed published from India in 2007, a twofold increase from 2002 (Table 1) - a 3.1 times increase for public health, 2.4 times for basic research and 1.5 times for clinical research. The sub-category clinical epidemiology had the highest relative increase. Within public health, the increase was lowest for the health system/policy sub-category. Only 5% of the original health research papers were in the public health research category in 2007, whereas 59.1% were in basic research and 35.9% in clinical research.

**Table 1:** Distribution of categories of original health research papers from India included in PubMed.

**Type of health research**	**No. (%) of papers in 2002**	**No. (%) of papers in 2007**	**2007 to 2002 ratio**
***Basic research***	2227 (49.6)	5360 (59.1)	2.4

Pure	518 (11.5)	1897 (20.9)	3.7

Applied	1709 (38.0)	3463 (38.2)	2.0

***Clinical research***	2119 (47.2)	3253 (35.9)	1.5

Patient series/management	1639 (36.5)	1642 (18.1)	1.0

Laboratory studies/clinical investigations	277 (6.2)	915 (10.1)	3.3

Clinical trials	153 (3.4)	312 (3.4)	2.0

Clinical epidemiology	50 (1.1)	384 (4.2)	7.7

***Public health research***	148 (3.3)	453 (5.0)	3.1

Epidemiology	72 (1.6)	249 (2.7)	3.5

Behavioural/environmental/social	31 (0.7)	119 (1.3)	3.8

Health system/policy	45 (1.0)	85 (0.9)	1.9

**Total**	4494 (100)	9066 (100)	2.0

**Figure 1:**
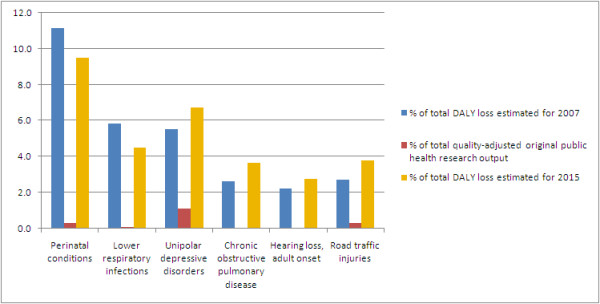
**Under-represented disease conditions in original public health research papers from India in 2007**. Conditions shown were estimated to contribute more than 2% of the total disability-adjusted life years (DALY) lost in India in 2007 and 2015, and had less than a third of the proportional original public health research output as compared with their contribution to disease burden.

Of the 453 original public health research papers in 2007, the majority were on epidemiology (55%), with 26.3% on behavioural, environmental or social aspects and 18.8% on health system or policy. In this last category, health information system and health policy were the most poorly represented sub-categories, covered by only 0.2% and 0.4% of the public health research papers. The proportion of public health research papers published in international journals, as compared with Indian journals, increased from 57.4% in 2002 to 74.8% in 2007. A comparison of quality-adjusted public health research output with the causes of disease burden revealed that there was a disproportionately low relative research published for several leading causes of disease burden (Figure 1). However, for ischaemic heart disease, which was estimated to contribute 5.6% of the total disease burden in India in 2007, the low 0.2% output of original quality-adjusted public health research in 2002 increased to a respectable 7.4% in 2007. The number of original public health research papers on the evaluation of population health interventions or policies increased from six in 2002 to 21 in 2007; this was still only 4.6% of the public health papers and 0.2% of all original health research papers. There were no papers on the evaluation of population interventions for the leading non-communicable causes of disease burden in India, such as cardiovascular disease, major depression and chronic obstructive pulmonary disease, or on road traffic injuries, and neither were there any on lower respiratory tract infections or tuberculosis which are among the leading communicable causes of disease burden in India.

The largest share of the 453 original public health research papers from India in 2007 was produced by medical and paramedical academic institutions (51.7%), followed by institutions under the Indian Council of Medical Research (13.9%), university departments other than health (13.7%), and foundations or other not-for-profit non-governmental organizations (10.6%). This trend was similar to that observed for the public health research papers from India in 2002 [1].

### Reports on public health research

We identified a total of 540 original public health research reports produced by institutions based in India from 2001 to 2008 (Additional file 2). There were 336 reports produced during 2005-2008, a 64.7% increase from the 204 reports produced during 2001-2004 (Table 2). Among disease/health conditions, the highest proportions of reports were on reproductive and child health (34.3%) and HIV/AIDS (13.3%). The proportion of reports on reproductive and child health decreased slightly from 38.7% in 2001-2004 to 31.5% in 2005-2008 (*P *= 0.09), while the proportion on the leading chronic non-communicable conditions (cardiovascular disease, diabetes, stroke, cancer, mental health and blindness) and injuries increased from 6.4% to 13.4% (*P *= 0.01). In 27.4% of the reports, health system/policy issues were the main topic without focus on specific disease/health conditions. This proportion was similar in the two 4-year blocks and included health policy or governance (10.0%) and health services (7.4%) as the highest proportions and health information system as the lowest proportion (0.6%). Environmental, social and development aspects of health comprised 7.4% of the reports, with the same proportions in the two 4-year blocks. Of the total reports in 2001-2004 and 2005-2008, 26% and 31.5%, respectively, were evaluations (*P *= 0.17). Of the 180 reports in 2001-2004 that covered specific states, 57.2% included one or more states from the group of 16 underdeveloped states that comprise half of India's population; this proportion was 58.9% of the 285 similar reports in 2005-2008.

**Table 2:** Health conditions, health system components and other health issues covered by original public health research reports from India, 2001-2008.

**Topics covered by reports**	**No. (%) of reports in 2001-2004**	**No. (%) of reports in 2005-2008**	**Total no. (%) of reports in 2001-2008**	**No. (%) of reports that were evaluations**
***Health conditions corresponding to national health programmes or themes***				

Reproductive and child health	79 (38.7)	106 (31.5)	185 (34.3)	79 (42.7)

HIV/AIDS	26 (12.7)	46 (13.7)	72 (13.3)	17 (23.6)

Diabetes and cardiovascular disease	5 (2.5)	18 (5.4)	23 (4.3)	5 (21.7)

Tuberculosis	5 (2.5)	6 (1.8)	11 (2.0)	10 (90.9)

Cancer	4 (2.0)	7 (2.1)	11 (2.0)	0

Injury	3 (1.5)	13 (3.9)	16 (3.0)	4 (25.0)

Vector borne diseases*	3 (1.5)	3 (0.9)	6 (1.1)	1 (16.7)

Mental health	0	5 (1.5)	5 (0.9)	1 (20.0)

Blindness	1 (0.5)	2 (0.6)	3 (0.6)	1 (33.3)

Leprosy	0	3 (0.9)	3 (0.6)	2 (66.7)

Iodine deficiency disorders	0	1 (0.3)	1 (0.2)	1 (100)

Deafness	0	0	0	0

Others†	5 (2.5)	9 (2.7)	14 (2.6)	5 (35.7)

***Health system components*‡**				

Health policy/governance§	19 (9.3)	35 (10.4)	54 (10.0)	11 (20.4)

Health services||	21 (10.3)	19 (5.7)	40 (7.4)	8 (20.0)

Health economics/financing	9 (4.4)	16 (4.8)	25 (4.6)	1 (4.0)

Human resources/training	7 (3.4)	13 (3.9)	20 (3.7)	3 (15.0)

Medical products/technologies¶	0	6 (1.8)	6 (1.1)	1 (16.7)

Health information system	1 (0.5)	2 (0.6)	3 (0.6)	1 (33.3)

***Other major health themes*‡**				

Environmental health**	11 (5.4)	9 (2.7)	20 (3.7)	7 (35.0)

Social determinants of health††	4 (2.0)	11 (3.3)	15 (2.8)	1 (6.7)

Development and health	0	5 (1.5)	5 (0.9)	0

Mortality and life expectancy	1 (0.5)	1 (0.3)	2 (0.4)	0

**Total (%)**	**204 (100)**	**336 (100)**	**540 (100)**	**159 (29.4)**

*These six reports included two on chikungunya, one on malaria, one on lymphatic filariasis, one on malaria, kala-azar and Japanese encephalitis together, and one on malaria, filariasis and dengue together.

†These 14 reports included six on oral health, four on disability, two on nutrition in general population, one on musculoskeletal conditions and one on gallbladder disease.

‡These reports were not on specific health conditions.

§These 54 reports included 26 on health system development/reform policies, nine on governance, nine on pharmaceutical policies, four on policies addressing vulnerable groups, three on food safety/security policies, two on policies for poverty reduction and health improvement, and one on health care waste management policy.

||Of these 40 reports, 25 covered both public and for-profit private health services, 11 covered public services only, three covered for-profit private services only and one covered not-for-profit private services only.

¶All six reports related to medicines/pharmaceuticals.

**These reports included air pollution and water and sanitation issues.

††These reports included living conditions, ageing, gender, migration and education issues.

**Figure 2:**
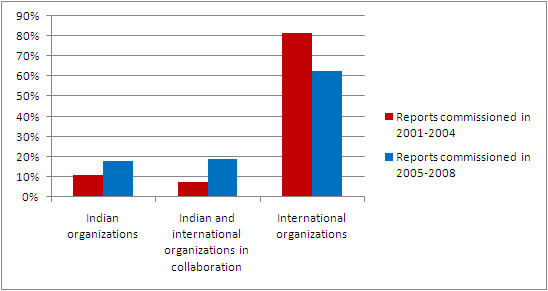
**Distribution of organizations that commissioned original public health research reports in India, 2001-2008**.

Of the total 540 reports, 304 (56.3%) were commissioned, with similar proportions commissioned among the reports in 2001-2004 and 2005-2008. Of the commissioned reports 37.2% were evaluations, compared to 19.5% of the non-commissioned reports (*P *< 0.001). The proportion of reports commissioned by the Indian governmental organizations, alone or in collaboration with international organizations, increased from 18.5% in 2001-2004 to 37.1% in 2005-2008 (*P *< 0.001), with a corresponding drop in reports commissioned exclusively by international organizations, although this still constituted 62.9% of the commissioned reports (Figure 2). Of the 533 reports that mentioned the organization(s) which conducted the research, 62.3% were by Indian organizations and 16.3% by Indian organizations in collaboration with international organizations (Table 3). The proportion of which research was done exclusively by international organizations based in India remained similar in 2001-2004 (22.5%) and 2005-2008 (20.7%). Of the research reports by Indian organizations, the largest proportion was by not-for-profit non-governmental research institutions and the central Indian government ministries and agencies, followed by university departments other than health and the medical and paramedical academic institutions, with a small proportion by for-profit private organizations. Of the research reports by international organizations, the largest number was by multilateral organizations. A greater proportion of reports by international organizations were evaluations (48.2%) compared with those by Indian organizations (22%) or those by collaborations between Indian and international organizations (29.9%); (*P *< 0.001).

**Table 3:** Organizations that produced the original public health research reports from India, 2001-2008.

**Organizations that produced the reports**	**No. (%) of reports in 2001-2004**	No. (%) of reports in 2005-2008*	Total no. (%) of reports in 2001-2008*
***Indian organizations***	**119 (58.3)**	**213 (64.7)**	**332 (62.3)**

Government organizations	42 (20.6)	56 (17.0)	98 (18.4)

Central Ministry of Health and its agencies	28 (13.7)	33 (10.0)	61 (11.4)

*ICMR institutes*†	*9 (4.4)*	*14 (4.3)*	*23 (4.3)*

Other central Ministries and government agencies	14 (6.9)	22 (6.7)	36 (6.8)

*ICSSR institutes*‡	*9 (4.4)*	*11 (3.3)*	*20 (3.8)*

State or local government agencies	0	1 (0.3)	1 (0.2)

University departments§	23 (11.3)	37 (11.2)	60 (11.3)

Not-for-profit health research institutions	19 (9.3)	22 (6.7)	41 (7.7)

Not-for-profit development and economics research institutions	6 (2.9)	19 (5.8)	25 (4.7)

Other not-for-profit research institutions	11 (5.4)	34 (10.3)	45 (8.4)

Medical & paramedical academic institutions	6 (2.9)	25 (7.6)	31 (5.8)

For-profit private organizations	8 (3.9)	13 (4.0)	21 (3.9)

Collaborations between Indian organizations	4 (2.0)	7 (2.1)	11 (2.1)

***International organizations with base in India***	**46 (22.5)**	**68 (20.7)**	**114 (21.4)**

Multilateral organizations||	24 (11.8)	26 (7.9)	50 (9.4)

Bilateral organizations¶	5 (2.5)	6 (1.8)	11 (2.1)

Others**	11 (5.4)	31 (9.4)	42 (7.9)

Collaborations between international organizations	6 (2.9)	5 (1.5)	11 (2.1)

***Collaborations between Indian and international organizations***	**39 (19.1)**	**48 (14.6)**	**87 (16.3)**

Indian government organizations and international organizations	7 (3.4)	21 (6.4)	28 (5.3)

Other Indian organizations and international organizations	32 (15.7)	27 (8.2)	59 (11.1)

**Total (%)**	**204 (100)**	**329 (100)**	**533 (100)**

*Seven reports not included here as these did not mention the organization that produced the report or authors.

†Indian Council of Medical Research (ICMR) has a nationwide network of institutes and is part of the Ministry of Health.

‡Indian Council of Social Science Research (ICSSR) has a nationwide network of institutes and is part of the Ministry of Human Resource Development.

§Departments other than medical or paramedical.

||Multilateral organizations included Joint United Nations Programme on HIV/AIDS, United Nations Development Fund for Women, United Nations Environmental Programme, United Nations Development Programme, World Bank and World Health Organization.

¶Bilateral organizations included German Technical Cooperation, Swedish International Development Cooperation Agency, and United States Agency for International Development.

**This included a variety of international organizations other than multilateral and bilateral organizations.

**Figure 3:**
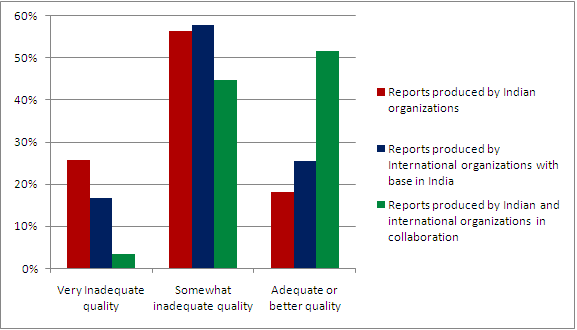
**Quality distribution of original public health research reports by organizations producing the reports, 2001-2008**. Reports with a quality score of 33% or less were considered to be of very inadequate quality, those with a score of 34-66% were considered somewhat inadequate and those with a score of 67% or more were considered to be of adequate or better quality.

Of the total 540 reports, 20.6% had a very inadequate quality score, 54.4% had a somewhat inadequate quality score and 25% had an adequate or better quality score. The distribution of quality was similar for reports produced in 2001-2004 and 2005-2008. Reports on disease/health conditions had a slightly higher proportion with adequate or better quality score (27.4%) compared to reports on health system or other health themes (20.5%); (*P *= 0.08). Reports that were commissioned included 32.2% with adequate or better quality score compared with 15.7% among non-commissioned reports (*P *< 0.001). The quality of reports produced by collaborations between Indian and international organizations was higher compared to those produced by Indian or international organizations alone, with 51.7% among the former group receiving an adequate or better quality score (*P *< 0.001) (Figure 3).

## Discussion

India has the greatest total disease burden of any country in the world [2]. Relevant public health research is fundamental to the reduction this burden. In this paper, the analysis of original public health research output from India during 2001-2008 reveals important trends and gaps that can inform further development of public health research capacity in India. A major advantage of this analysis is the inclusion of research reports in addition to published papers, as research reports play a significant role in the development of health programmes and policies.

The contribution of all papers from India in PubMed as a proportion of the global total increased by about 50% - from 1.1% in 2002 to 1.6% in 2007. The three-fold increase from 2002 to 2007 in original public health research papers included in PubMed versus an overall doubling of the number of original health research papers is encouraging. However, the proportion of public health research papers was still low, at 5% of the total health research papers from India in 2007. In comparison, 12% of the health research papers from Australia included in PubMed in 2002 were on public health [1]. In addition to the low relative proportion of public health research papers from India, the distribution of topics covered continued to have a number of deficiencies. Under-representation of many major causes of disease burden, health system research and evaluations of population health interventions was still present in 2007. These continuing major deficits suggest that a more focused effort is necessary to steer research publications emanating from India to adequately represent the major causes of disease burden and critical health system issues.

The 65% increase from 2001-2004 to 2005-2008 in the original public health research reports produced from India, which were identified in the public domain through internet search, could be due to an actual increase in production and/or a higher likelihood of later reports to be placed in the electronic form on websites. The over-representation of reports on reproductive and child health and on HIV/AIDS is probably related to the very high profile and huge international funding of these two health programmes in India. In contrast, however, perinatal conditions were grossly underrepresented in the published papers in PubMed. There was some increase in the number of reports on the leading chronic non-communicable diseases and injuries from 2001-2004 to 2005-2008, but this still comprised only one-fifth of the health condition specific reports in 2005-2008 which is not commensurate with the estimated over half of the disease burden in India caused by these conditions [2,16,17].

Reports on health system and policy issues, which did not focus on any specific disease/health conditions, comprised 27% of all original public health research reports. Within this group, over one-third of the reports were on health policy or governance, which is encouraging. However, research on the health information system was the most poorly represented topic in this group. This is an ominous trend as a strong health information system is a fundamental requirement for the improvement of population health [12,18]. Only 7% of the total reports were on the environmental, social and development aspects of health. Given the increasing realization of the major influence that social and environmental determinants exert on health [19,20], research on these aspects needs more attention in India. An encouraging finding was that the group of 16 underdeveloped states, which comprise half of India's population, was adequately represented in the reports.

About 30% of the original public health research reports were evaluations, a much higher proportion than seen in the original public health research papers in PubMed from India. The vast majority of reports on tuberculosis and just under half of the reports on reproductive and child health were evaluations. These two conditions have long-standing health programmes in India. The high proportion of evaluation reports among these conditions could be related to an interest in knowing the outcome and impact of programmes that have been receiving investments over a long period of time. There was a suggestion of an increase from 2001-2004 to 2005-2008 in the proportion of total reports that were evaluations. This is a good sign as evaluations are crucial to understand how well society's resources are being utilized. However, evaluations were twice as common among reports produced by international organizations, suggesting that evaluations need more emphasis among Indian organizations.

About half of the original identified original public health research reports produced in 2001-2004 and 2005-2008 had been commissioned. The proportion of reports commissioned by Indian governmental organizations alone, or in collaboration with international organizations, doubled from 2001-2004 to 2005-2008. This is an important trend which could be due to an increasing realization among Indian government agencies that they need to encourage public health research. Perhaps not surprisingly, evaluations were twice as common among commissioned reports as among non-commissioned reports.

The quality of original public health research reports revealed much to be desired. Only one in four reports were scored as being of an adequate or better quality. Reports on specific health conditions were of a slightly higher quality than reports on the health system or other health themes, suggesting that relatively higher attention is needed towards the latter. Commissioned reports were twice as likely to be of adequate or better quality as compared with non-commissioned reports, which could be related to a higher perceived accountability for commissioned reports. Reports produced by Indian and international organizations in collaboration were two and a half times more likely to be of adequate or better quality than those produce by Indian or international organizations alone. This important finding highlights the value of combining skills and perspectives in order to produce higher quality research and suggests that it would be useful to encourage further such collaborations in India.

About half of the published original public health research papers from India in PubMed in 2007 were produced by medical and paramedical academic institutions, about an eighth by the Indian Council of Medical Research institutions and university departments other than health, and about a tenth by not-for-profit non-governmental organizations. In contrast, over one-third of the original public health research reports produced by Indian organizations during 2001-2008 were by not-for-profit non-governmental research institutions, a quarter by central Indian government ministries and agencies, about a sixth by university departments other than health, and about an eighth by medical and paramedical academic institutions. This diverse distribution of organizations in India producing public health research in the form of papers and reports indicates that systematic efforts to enhance long-term public health research capacity in India would require the involvement of this range of stakeholders. National level organizations in India that could potentially help coordinate this effort include the Department of Health Research which was established in 2007 by the Ministry of Health in India to respond to national health priorities [21], and the Public Health Foundation of India which was launched by the Prime Minister of India in 2006 to strengthen public health training and research in India [7].

The availability of appropriate funding is a pre-requisite for conducting relevant public health research in India. The financing architecture of health research in India is not well documented. Estimates by the National Health Accounts of India and the Global Forum for Health Research suggest that about US$225 million was spent on health research and development in India in 2001, which was about 1% of the total health expenditure and about 0.05% of the gross domestic product of India [22,23]. This included 63% in the private for-profit pharmaceutical sector, 26% through the central Health Ministry, 4% through other central ministries and agencies, 3% through state governments and 4% by not-for-profit international organizations. Funding for health research in India has increased substantially over the past few years from both domestic and international sources. The budget for the Indian Council for Medical Research, which forms a big portion of the central Health Ministry research spending, has more than tripled from 2001 to 2008 [24]. Major funding was announced in 2008 by the UK-based Wellcome Trust for a Biomedical Research Career Programme for India jointly funded by the Government of India's Department of Biotechnology and three strategic awards for public health research and capacity building in India [25]. The increase in the outsourcing of clinical trials of drugs to India is also bringing in more funding but is also posing some challenges [26]. While more funding is becoming available for health research in India from various sources, the entire range of health research funding and a composite picture of what it is being spent on are not clearly understood. A tracking mechanism is needed that could guide the best use of funding commensurate with the evolving disease and health system priorities, including appropriate emphasis on developing the neglected aspects of public health research in India.

There are some limitations of our analysis. First, we only included published research output from the PubMed database for a standardized comparison with our previous analysis of research output from India [1]. The PubMed database continues to be used for bibliometric analysis of research output from different parts of the world [27-31]. While some health publications from India that did not qualify for inclusion in PubMed would have been missed in our analysis, it is unlikely that this would have affected the observed time-trends in a major way as PubMed is one of the most comprehensive bibliographic databases for health literature. A useful subsequent addition to this analysis would be review of trends in social science bibliographic databases for health-related research output from India. Second, in our analysis we included reports on original public health research that were available on the internet. Ready availability of reports in the public domain on the internet enables widespread use of their findings, and we were interested in capturing the trends in such reports as we believe that this is the most relevant trend to be understood in the first instance. We attempted extensive internet searches, but we may have still missed some reports. However, it seems unlikely that a few missed reports would have altered the major findings of our review of reports. Third, our quality assessment criteria for the reports were based on our understanding of what is desirable in public health research reports. There could certainly be other criteria for assessing quality, but we believe that the broad patterns of quality of public health research reports that we found are a useful start for probing this issue further in India.

The analytical approach used in this paper to assess public health research output and its relation with the disease burden and health system priorities in India could also be useful for other developing countries.

## Conclusion

The combination of findings in this paper from published papers and reports readily available in the public domain define the major trends of public health research in India since 2001. These findings show an increase in the availability of public health research output from India over the past few years, which is encouraging, but also highlight that the quality of research produced is not satisfactory, the distribution of research topics continues to be inconsistent with the disease burden trends and some major health system components such as the health information system are being overlooked. These findings have substantial implications for health policy in India. Acute attention is needed to address the identified continuing major deficits in public health research in order for India to more effectively reduce its very large disease burden. The trends and gaps in the research output, and the organizations involved in its production, reported in this paper offer valuable pointers to the development of a framework for enhancing the public health research capacity, infrastructure and resources in India.

## Competing interests

The authors declare that they have no competing interests.

## Authors' contributions

LD led the design, analysis, interpretation and drafting of the manuscript. MZR accessed the public health research reports and reviewed them in collaboration with LD and RD, and contributed to the analysis and drafting of the manuscript. RKG accessed the PubMed publications and reviewed them under the guidance of LD and RD, and contributed to the analysis. AB contributed to the data collection and analysis of funding for health research in India in collaboration with LD. RD contributed to the design, analysis, interpretation and drafting of the manuscript. All authors reviewed and approved the manuscript.

## Pre-publication history

The pre-publication history for this paper can be accessed here:



## Supplementary Material

Additional file 1 **Websites searched for original public health research reports**. List of websites of organizations that were searched to identify original public health research reports produced during 2001-2008 from India. Click here for file

Additional file 2 **Identified original public health research reports**. List of identified original public health research reports produced during 2001-2008 from India that were available in the public domain on the internet. Click here for file
