# Supplementary material for: Trends of public health research output from India during 2001-2008
Source: BMC Med. 2009 Oct 14;7:59. doi: 10.1186/1741-7015-7-59 (PMC2766381; doi:10.1186/1741-7015-7-59)
Supplement: Additional file 1 — Websites searched for original public health research reports. List of websites of organizations that were searched to identify original public health research reports produced during 2001-2008 from India. [file 1741-7015-7-59-S1.DOC]

**Trends of Public Health Research Output from India 2001-2008**

Lalit Dandona, Magdalena Z. Raban, Rama K. Guggilla, Aarushi Bhatnagar, Rakhi Dandona

**Additional file 1:** Websites searched for original public health research reports.

This file lists the websites of organizations that were searched to identify original public health research reports produced during 2001-2008 from India.

**A. Indian organizations**

**National Ministry of Health**

**Ministry of Health and Family Welfare, New Delhi**

- ASHA Accredited Social Health Activist (<http://www.mohfw.nic.in/NRHM/asha.htm>)
- Central Bureau of Health Intelligence (<http://www.cbhidghs.nic.in/>)
- Department of AYUSH (<http://indianmedicine.nic.in/systemwise-statistics.asp>)
- Directorate General of Health Services (<http://mohfw.nic.in/dghsindex.htm>)
- Ministry of Health and Family Welfare (<http://mohfw.nic.in/>)
- National Aids Control Organisation (<http://www.nacoonline.org/NACO>)
- National Commission on Population ([http://www.populationcommission.nic.in](http://www.populationcommission.nic.in/))
- National Health Information Collaboration (<http://www.nhicindia.org/Type.asp>)
- National Health Programmes (<http://mohfw.nic.in/healthprogmain.html>)
- National Health Systems Resource Centre (<http://nhsrcindia.org/index.php?option=com_contact&view=contact&id=2&Itemid=64>)
- National Institute of Communicable Diseases (<http://nicd.nic.in/Idownload.asp>)
- National Institute of Health and Family Welfare (<http://www.nihfw.org/index.asp>)
- National Iodine Deficiency Disorders Control Programme (<http://www.mohfw.nic.in/NRHM/niddcp.htm>)
- National Leprosy Eradication Programme (<http://nlep.nic.in/>)
- National Mental Health Programme ([http://mohfw.nic.in/Mental%20Health.pdf](http://mohfw.nic.in/Mental Health.pdf))
- National Polio Surveillance Project (<http://www.npspindia.org/>)
- National Programme for Prevention and Control of Diabetes, Cardiovascular Disease and Stroke (<http://mohfw.nic.in/NPDCS.htm>)
- National Programme for the Control of Blindness (<http://mohfw.nic.in/default.htm>)
- National Programme for the Control of Deafness ([http://mohfw.nic.in/Website/Contents%20of%20Website.htm](http://mohfw.nic.in/Website/Contents of Website.htm))
- National Rural Health Mission (<http://mohfw.nic.in/NRHM.htm>)
- National Tobacco Control Programme ([http://mohfw.nic.in/National%20Programme%20for%20Tobacco%20Control.htm](http://mohfw.nic.in/National Programme for Tobacco Control.htm))
- National Vector Borne Disease Control Programme (<http://nvbdcp.gov.in/>)
- Reports and publications page (<http://mohfw.nic.in/index.htm>)
- Reproductive and Child Health Phase-II (<http://www.mohfw.nic.in/NRHM/RCH/Index.htm>)
- Tuberculosis Control in India (<http://www.tbcindia.org/RNTCP.asp>)

**Indian Council of Medical Research**

- Centre for Research in Medical Entomology, Madurai (<http://icmr.nic.in/pinstitute/crme.htm>)
- Desert Medicine Research Centre Jodhpur (<http://www.dmrcjodhpur.org/>)
- Enterovirus Research Centre, Mumbai (<http://icmr.nic.in/pinstitute/evrc.htm>)
- Food and Drug Toxicology Research Centre, Hyderabad (<http://www.ninindia.org/>)
- Genetic Research Centre, Mumbai (<http://icmr.nic.in/pinstitute/grc/grc.htm>)
- Indian Council of Medical Research, New Delhi (<http://icmr.nic.in/>)
- Institute of Cytology and Preventive Oncology, Noida (<http://www.icpo.org.in/>)
- Institute of Pathology, New Delhi (<http://icmr.nic.in/pinstitute/iop.htm>)
- Microbial Containment Complex, Pune (<http://icmr.nic.in/pinstitute/mcc.htm>)
- National AIDS Research Institute, Pune (<http://www.nari-icmr.res.in/>)
- National Cancer Registry Programme, Bangalore (<http://icmr.nic.in/ncrp/cancer_reg.htm>)
- National Centre for Laboratory Animal Science, Hyderabad (<http://www.ninindia.org/>)
- National Institute for Research in Reproductive Health, Mumbai (<http://icmr.nic.in/pinstitute/irr.htm>)
- National Institute of Cholera and Enteric Diseases, Kolkata (<http://www.niced.org/>)
- National Institute of Epidemiology, Chennai (<http://www.nie.gov.in/>)
- National Institute of Immunohaematology, Mumbai (<http://icmr.nic.in/pinstitute/iih.htm>)
- National Institute of Malaria Research, New Delhi (<http://www.mrcindia.org/>)
- National Institute of Medical Statistics, New Delhi (<http://icmr.nic.in/pinstitute/nims.htm>)
- National Institute of Nutrition, Hyderabad (<http://www.ninindia.org/>)
- National Institute of Occupational Health, Ahmedabad (<http://www.nioh.org/>)
- National Institute of Virology, Pune (<http://www.niv.co.in/>)
- National JALMA Institute for Leprosy & Other Mycobacterial Diseases, Agra (<http://www.jalma-icmr.org.in/>)
- National Nutrition Monitoring Bureau, Hyderabad (<http://www.nnmbindia.org/>)
- Rajendra Memorial Research Institute of Medical Sciences, Patna (<http://www.rmrims.org.in/>)
- Regional Medical Research Centre, Belgaum (<http://icmr.nic.in/pinstitute/belgaum.htm>)
- Regional Medical Research Centre, Bhubaneswar (<http://icmr.nic.in/pinstitute/bhubaneswar.htm>)
- Regional Medical Research Centre, Dibrugarh (<http://rmrcne.org/>)
- Regional Medical Research Centre, Jabalpur (<http://www.rmrct.org/>)
- Regional Medical Research Centre, Port Blair (<http://www.rmrc.res.in/default.htm>)
- Tuberculosis Research Centre, Chennai (<http://www.trc-chennai.org/>)
- Vector Control Research Centre, Puducherry (<http://vcrc.res.in/>)

**Other National Ministries or Government Agencies**

- Bharat Nirman (<http://bharatnirman.gov.in/page2.html>)
- Census of India ([http://www.censusindia.gov.in](http://www.censusindia.gov.in/))
- Central Pollution Control Board (<http://www.cpcb.nic.in/>)
- Council for Advancement of People’s Action and Rural Technology (<http://capart.nic.in/pub/index.html>)
- Education for All in India (<http://www.educationforallinindia.com/>)
- Ministry of Agriculture (<http://agricoop.nic.in/progs.htm>)
- Ministry of Consumer Affairs, Food and Public Distribution (<http://fcamin.nic.in/>)
- Ministry of Development of North Eastern Region (<http://mdoner.gov.in/index2.asp?sid=135>)
- Ministry of Environment and Forests (<http://envfor.nic.in/>)
- Ministry of Finance (<http://finmin.nic.in/reports/index.html>)
- Ministry of Heavy Industry and Public Enterprises (<http://www.dhi.nic.in/> and <http://dpe.nic.in/>)
- Ministry of Home Affairs (<http://www.mha.nic.in/>)
- Ministry of Housing and Urban Poverty Alleviation (<http://mhupa.gov.in/>)
- Ministry of Human Resource Development
- Ministry of Labour and Employment (<http://labour.nic.in/>)
- Ministry of Micro, Small and Medium Enterprises (<http://msme.gov.in/msme_publications.htm>)
- Ministry of Minority Affairs (<http://minorityaffairs.gov.in/newsite/index.asp>)
- Ministry of Panchayati Raj (<http://panchayat.gov.in/viewPortalPage.do?PPID=200>)
- Ministry of Rural Development (<http://www.rural.nic.in/>)
- Ministry of Shipping, Road Transport and Highways (<http://cabsec.nic.in/abr/abr29c.htm>)
- Ministry of Social Justice and Empowerment (<http://socialjustice.nic.in/publication.html>)
- Ministry of Statistics and Programme Implementation (<http://mospi.nic.in/>)
- Ministry of Tribal Affairs (<http://tribal.nic.in/index1.html>)
- Ministry of Urban Development (<http://urbanindia.nic.in/moud/programme/ud/main.htm>)
- Ministry of Water Resources (<http://wrmin.nic.in/index2.asp?slid=795&sublinkid=580&langid=1>)
- Ministry of Women and Child Development (<http://wcd.nic.in/>)
- Ministry of Youth Affairs and Sports (<http://yas.nic.in/index.asp?layid=1>)
- National Human Rights Commission (<http://nhrc.nic.in/publications.htm>)
- National Sample Survey Organisation (<http://mospi.nic.in/nsso_test1.htm>)
- Planning Commission (<http://planningcommission.nic.in/>)
- Pradhan Mantri Gram Sadak Yojana (PMGSY) (<http://pmgsy.nic.in/>)

**Indian Council of Social Science Research**

- Centre for Development Studies, Thiruvananthapuram (<http://www.cds.edu/>)
- Centre for Economic and Social Studies, Hyderabad (<http://www.cess.ac.in/cesshome/cessmain.asp>)
- Centre for Multi-Disciplinary Development Research, Dharwad (<http://www.cmdr.co.in/index.htm>)
- Centre for Policy Research, New Delhi (<http://www.cprindia.org/index.php>)
- Centre for Research in Rural and Industrial Development, Chandigarh (<http://www.crrid.res.in/>)
- Centre for Social Studies, Surat (<http://www.css.ac.in/>)
- Centre for Studies in Social Sciences, Kolkata (<http://www.cssscal.org/>)
- Centre for Studies in Social Sciences, Kolkata (<http://www.cssscal.org/>)
- Centre for the Study of Developing Societies, New Delhi (<http://www.csds.in/index.php>)
- Centre for Women’s Development Studies, New Delhi (<http://www.cwds.org/publications.htm>)
- Council for Social Development, Hyderabad (<http://www.csdindia.org/>)
- Dr. Babasaheb Ambedkar National Institute of Social Sciences, Dongargaon (<http://www.baniss.org/>)
- G.B. Pant Social Science Institute, Allahabad (<http://gbpssi.nic.in/index.htm>)
- Gandhian Institute of Studies, Varanasi
- Giri Institute of Development Studies, Lucknow (<http://www.gids.org.in/index.asp>)
- Gujarat Institute for Development Research, Ahmedabad
- Indian Council of Social Science Research (<http://www.icssr.org/>)
- Indian Institute of Education, Pune (<http://www.iiepune.org/html/aboutus.htm>)
- Institute for Social and Economic Change, Bangalore (<http://www.isec.ac.in/>)
- Institute for Studies in Industrial Development, New Delhi (<http://isid.org.in/>)
- Institute of Development Studies, Jaipur (<http://www.idsj.org/>)
- Institute of Public Enterprise, Hyderabad (<http://www.ipeindia.org/>)
- Madhya Pradesh Institute of Social Science Research, Ujjain (<http://www.mpissr.org/>)
- Madras Institute of Development Studies, Chennai (<http://www.mids.ac.in/aboutus.htm>)
- Nabakrushna Choudhury Centre for Development Studies, Bhubaneshwar, Orissa (<http://nkccds.nic.in/aboutus.asp>)
- Omeo Kumar Das Institute of Social Change and Development, Guwahati (<http://www.okd.in/index.html>)
- Sardar Patel Institute of Social and Economic Research, Ahmedabad (<http://www.spiesr.ac.in/index.htm>)

**Academic and research institutions**

- Administrative Staff College of India, Hyderabad (<http://www.asci.org.in/>)
- All India Institute of Medical Sciences, New Delhi (<http://www.aiims.edu/>)
- Armed Forces Medical College, Pune (<http://afmc.nic.in/>)
- Centre for Chronic Disease Control India, New Delhi (<http://www.ccdcindia.org/>)
- Centre for Enquiry into Health and Allied Themes, Mumbai (<http://www.cehat.org/go/>)
- Centre for Social Science Medicine, Jawaharlal Nehru University, New Delhi (<http://www.jnu.ac.in/main.asp?sendval=SchoolOfSocialSciences>)
- Christian Medical College, Vellore (<http://cmch-vellore.edu/main.asp>)
- Grant Medical College and Sir J.J. Group of Hospitals, Mumbai (<http://www.grantmedicalcollege-jjhospital.org/>) Indian Institute of Health Management Research, Jaipur (<http://www.iihmr.org/>)
- Indian Institute of Management, Ahmedabad (<http://www.iimahd.ernet.in/>)
- Indian Institute of Management, Bangalore (<http://www.iimb.ernet.in/>)
- Institute for Health Systems, Hyderabad (<http://www.ihs.org.in/index.htm>)
- Institute for Economic Growth, New Delhi (<http://www.iegindia.org/>)
- Institute of Public Health, Bangalore (<http://www.iphindia.org/new/index.php?option=com_content&task=view&id=61&Itemid=61>)
- International Institute of Population Sciences, Mumbai (<http://www.iipsindia.org/>)
- International Institute of Population Sciences – District Level Household & Facility Survey (RCH Project), Mumbai (<http://www.rchiips.org/>)
- IndiaCLEN Program Evaluation Network (<http://www.ipen.org.in/>)
- Jawaharlal Institute of Postgraduate Medical Education and Research, Puducherry (<http://www.jipmer.edu/>)
- Kasturba Medical College, Mangalore (<http://www.manipal.edu/manipalsite/Users/colpage.aspx?collegeid=2&Id=1>)
- Kasturba Medical College, Manipal (<http://www.manipal.edu/manipalsite/Users/colpage.aspx?collegeid=1&Id=1>)
- King Edward Memorial Hospital and Seth Gordhandas Sunderdas Medical College, Mumbai (<http://www.kem.edu/>)
- Madras Medical College, Chennai (<http://www.mmcindia.net/>)
- Maulana Azad Medical College, New Delhi (<http://www.mamc.ac.in/>)
- National Council of Applied Economic Research, New Delhi (<http://www.ncaer.org/governing.html>)
- National Institute of Public Finance and Policy, New Delhi (<http://www.nipfp.org.in/aboutus.asp>)
- Pandit Bhagwat Dayal Sharma Post Graduate Institute of Medical Sciences, Rohtak (<http://www.pgims.org/>)
- Population Foundation of India, New Delhi (<http://www.popfound.org/>)
- Postgraduate Institute of Medical Education and Research, Chandigarh (<http://pgimer.nic.in/>)
- Pravara Institute of Medical Sciences (Deemed University), Loni (<http://www.pravara.com/research.html>)
- Public Health Foundation of India, New Delhi (<http://www.phfi.org/>)
- Sanjay Gandhi Post Graduate Institute of Medical Sciences, Lucknow (<http://www.sgpgi.ac.in/>)
- SEARCH, Gadchiroli (<http://www.searchgadchiroli.org/>)
- Social Watch India (<http://www.socialwatchindia.net/Index.asp>)
- Sree Chitra Tirunal Institute for Medical Sciences and Technology, Thiruvananthapurum(<http://www.sctimst.ac.in/amchss/publications/index.htm>)
- St John’s National Academy of Health Sciences, Bangalore (<http://www.stjohns.in/>)
- TATA Institute of Social Sciences, Mumbai(<http://www.tiss.edu/>)

**B. International organizations with operations/base in India**

**Bilateral**

- Canadian International Development Agency (<http://www.acdi-cida.gc.ca/index-e.htm>)
- Delegation of the European Commission to India (<http://www.delind.ec.europa.eu/en/index.htm>)
- Department for International Deveopment, U.K. (<http://www.dfid.gov.uk/>)
- Norway India Partnership Initiative (<http://www.norwayemb.org.in/development/NIPI/About%2BNIPI/nipi1.htm>)
- Norwegian Agency for Development Cooperation (NORAD) (<http://norad.no/en/Frontpage>)
- Swedish International Development Cooperation Agency (<http://www.sida.org/>)
- United States Agency for International Development (<http://www.usaid.gov/>)

**Multilateral**

- Asian Development Bank (<http://www.adb.org/Publications/country.asp?s=1&id=12&wp=1&p=ctryind>)
- Joint United National Programme on HIV/AIDS (UNAIDS) (<http://www.unaids.org/en/default.asp>)
- United Nations Children’s Fund (UNICEF) - India (<http://www.unicef.org/india/>)
- United Nations Development Fund for Women (UNIFEM) (<http://www.unifem.org.in/index.html>)
- United Nations Development Programme - India (<http://www.undp.org.in/>)
- United Nations Educational Scientific and Cultural Organization (UNESCO) (<http://whc.unesco.org/en/statesparties/in>)
- United Nations High Commissioner for Refugees (<http://www.unhcr.org/cgi-bin/texis/vtx/home>)
- United Nations Population Fund (UNFPA)(<http://india.unfpa.org/>)
- World Bank (<http://www.worldbank.org/>)
- World Health Organization (<http://www.who.int/>)
- World Health Organization India Country Office (<http://www.whoindia.org/EN/Index.htm>)
- World Health Organization South East Asia Regional Office (<http://www.searo.who.int/>)

**Others**

- Action Aid (<http://www.actionaid.org/india/index.aspx>)
- Aga Khan (<http://www.akdn.org/india_health.asp>)
- America India Foundation (<http://www.aif.org/default.htm>)
- Bill and Melinda Gates Foundation (<http://www.gatesfoundation.org/Pages/home.aspx>)
- CARE (<http://careindia.org/ManageHome/Home.aspx>)
- Catholic Relief Services (<http://crs.org/>)
- Clinton Foundation (<http://www.clintonfoundation.org/>)
- EngenderHealth (<http://www.engenderhealth.org/index.php>)
- Family Health International (<http://www.fhi.org/en/CountryProfiles/India/index.htm>)
- Ford Foundation (<http://www.fordfound.org/regions/indianepalsrilanka/overview>)
- Health Action International (<http://www.haiap.org/publications/>)
- International Centre for Research on Women (<http://www.icrw.org/asia/>)
- MacArthur Foundation (<http://www.macfound.org/site/c.lkLXJ8MQKrH/b.3599935/k.66CA/MacArthur_Foundation_Home.htm>)
- Naz Foundation (<http://www.nazindia.org/index.htm>)
- Orbis (<http://www.orbis.org/Default.aspx?cid=5713&lang=1>)
- Oxfam (<http://www.oxfamindia.org/>)
- Packard Foundation (<http://www.packard.org/home.aspx>)
- Panos Institute (http://www.panossouthasia.org/index.asp?sname=HOME)
- Pathfinder International (<http://www.pathfind.org/site/PageServer>)
- Population Council (<http://www.popcouncil.org/asia/indiaRI.html>)
- Population Services International (<http://www.psi.org/where_we_work/india.html>) (<http://www.psi.org.in/>)
- Program for Appropriate Technology in Health (<http://www.path.org/>)
- Project Hope (<http://www.projecthopeindia.com/>)
- Rockefeller Foundation (<http://www.rockfound.org/index.shtml>)
- Save the Children (<http://www.savethechildren.in/index.html>)
- Tearfund (<http://www.tearfund.org/>)
- World Vision (<http://www.worldvisionindia.org/>)
