# Supplementary material for: Trends of public health research output from India during 2001-2008
Source: BMC Med. 2009 Oct 14;7:59. doi: 10.1186/1741-7015-7-59 (PMC2766381; doi:10.1186/1741-7015-7-59)
Supplement: Additional file 2 — Identified original public health research reports. List of identified original public health research reports produced during 2001-2008 from India that were available in the public domain on the internet. [file 1741-7015-7-59-S2.DOC]

**Trends of Public Health Research Output from India 2001-2008**

Lalit Dandona, Magdalena Z. Raban, Rama K. Guggilla, Aarushi Bhatnagar, Rakhi Dandona

**Additional file 1:** Identified original public health research reports.

This file lists the identified original public health research reports produced during 2001-2008 from India that were available in the public domain on the internet.

**2001**

1. Akbar S, Lvovsky K. Indoor air pollution: energy and health for the poor - India's National Program of Improved Cookstoves: A growing challenge. 2001. http://www-wds.worldbank.org/external/default/WDSContentServer/WDSP/IB/2004/05/14/000090341_20040514131915/Rendered/PDF/284360Indoor0air0no.05.pdf
2. Bharat S, Aggleton P, Tyrer P. India: HIV and AIDS-related discrimination, stigmatization and denial. 2001. http://pdf.usaid.gov/pdf_docs/PNACM522.pdf
3. Bhat R, Verma B, Reuben E. Hospital efficiency: An empirical analysis of district and grant-in-aid hospitals in Gujarat. 2001. http://www.iimahd.ernet.in/publications/data/2001-07-05RameshBhat.pdf
4. Das J, Joshi C. Evaluation of obligatory free medical care services by non-governmental hospitals in Delhi (2001-2002). 2001. http://www.nihfw.org/asp/ResearchStudies.asp?currentpage=3
5. Das N, Mishra V, Saha P. Does community access affect the use of health and family welfare services in rural India? 2001. http://pdf.usaid.gov/pdf_docs/PNACM115.pdf
6. Dasgupta P. Valuing the damages from water pollution in urban Delhi, India: A health production function approach. 2001. <http://www.iegindia.org/workpap/wp210.pdf>
7. Deodhar S. WTO Agreements on SPS and TBT: Implications for food quality issues. 2001. http://202.54.104.236/intranet/eip/whorep/uploads/H/H-WTO%20Agreements%20on%20SPS%20and%20TBT%20Implications%20for%20Food%20Quality%20Issues.pdf
8. Family Health International. Mapping of commercial sex access points and relevant service outlets in Maharashtra. 2001. http://www.fhi.org/en/HIVAIDS/pub/survreports/Mappavertprepstudyindia.htm
9. Family Health International, AVERT Society, Impact. Communication needs assessment in Maharashtra. 2001. http://pdf.usaid.gov/pdf_docs/PNADK766.pdf
10. Gulati S, Sharma S. Reproductive and Child Health status in India: District level analysis. 2001. http://www.iegindia.org/dispap/dis44.pdf
11. Gumber A. Hedging the health of the poor: the case for community financing in India. 2001. http://www-wds.worldbank.org/external/default/WDSContentServer/WDSP/IB/2004/05/18/000265513_20040518164420/Rendered/PDF/288750Gumber1Hedging0the0Health1whole.pdf
12. Gupta I, Dasgputa P, Sawhney M. Health of the elderly in India: Some aspects of vulnerabilty. 2001. http://www.iegindia.org/dispap/dis26.pdf
13. Hawkes S, Santhya K. Diverse realities: Understanding sexually transmitted infections and HIV in India. 2001. http://www.popcouncil.org/asia/indiaRI.html
14. Indian Council of Medical Research. Consolidated report of the population based cancer registries 1990-96. 2001. http://icmr.nic.in/ncrp/ncrp_p/cancer_reg.pdf
15. Indian Market Research Bureau, Family Health International. Health care provider survey Maharashtra. 2001. http://www.fhi.org/en/HIVAIDS/pub/survreports/HCPavertprepstudyindia.htm
16. International Institute of Population Sciences. Reproductive and Child Health Project Rapid Household Survey (Phase I & 2) India 1998-1999. 2001. http://www.rchiips.org/pdf/rch1/National_Report_RCH-1.pdf
17. Jakab M, Preker A, Krishnan C, Schneider P, Diop F, Jutting J, Gumber A, Ranson K, Supakankunti S. Social inclusion and financial protection through community financing: initial results from five household surveys. 2001. http://www-wds.worldbank.org/external/default/WDSContentServer/WDSP/IB/2004/05/19/000265513_20040519123047/Rendered/PDF/288840Jakab01Social0Inclusion1whole.pdf
18. Jalan J, Ravallion M. Does piped water reduce diarrhoea for children in rural India? 2001. http://www-wds.worldbank.org/external/default/WDSContentServer/WDSP/IB/2001/11/22/000094946_01091104014290/Rendered/PDF/multi0page.pdf
19. Johnston H, Ved R, Lyall N, Agarwal K. Postabortion complications and their management: a community assessment conducted in rural Uttar Pradesh, India -- final report. 2001. http://pdf.usaid.gov/pdf_docs/PNACT470.pdf
20. Lalitha N. TRIPS and pharmaceutical industry: Issues of strategic importance. 2001. http://www.gidr.ac.in/gidr/pdf/WP-128.pdf
21. Mahal A, Yazbeck A, Peters D, Ramana G. The poor and health service use in India. 2001. http://www-wds.worldbank.org/external/default/WDSContentServer/WDSP/IB/2004/05/21/000265513_20040521150443/Rendered/PDF/288910Mahal01The0Poor0and01whole.pdf
22. Mishra U, Ramanathan M. Delivery complications and determinants of caesarean section rates in India - An analysis of National Family Health Surveys 1992-93. 2001. http://www.cds.edu/download_files/wp314.pdf
23. Mittra P. A study on functioning of Health and Family Welfare Training Centres, Regional Training Centres and Divisional Training Centres (2001-2002). 2001. http://www.nihfw.org/asp/ResearchStudies.asp?currentpage=4
24. Murali I. Development of epidemiological services in a district (2001-2002). 2001. http://www.nihfw.org/asp/ResearchStudies.asp?currentpage=3
25. Nandraj S, Madhiwalla N, Sinha R, Jesani A. Women and health care in Mumbai: A study of morbidity, utilisation & expenditure on health care by households of the metropolis. 2001. http://www.cehat.org/go/uploads/MumbaiStudy/mumbaistudy.pdf
26. Nandraj S, Muraleedharan V, Baru R, Qadeer I, Priya R. Private health sector in India: Review & annotated bibliography. 2001. http://www.cehat.org/go/uploads/Rphsi/phsi.pdf
27. ORG Centre for Social Research, Family Health International. Behavioural surveillance survey in Maharashtra. 2001. http://www.fhi.org/en/HIVAIDS/pub/survreports/Bssavertprepstudyindia.htm
28. Planning Commission. Evaluation study on functioning of Primary Health Centres (PHCs) assisted under Social Safety Net Programme (SSNP). 2001. http://planningcommission.nic.in/reports/peoreport/peoevalu/peo_ssnp.pdf
29. Prabhu S & Selvaraju V. Public financing for health security in India: Issues and trends. 2001. http://nhsrcindia.org/index.php?option=com_docman&task=doc_view&gid=70&tmpl=component&format=raw&Itemid=115
30. Retherford R, Mishra V, Prakasam G. How much has fertility declined in Uttar Pradesh? 2001. http://pdf.usaid.gov/pdf_docs/PNACM114.pdf
31. Sankar D. The role of traditional and alternative health systems in providing health care options: Evidence from Kerala. 2001. http://www.iegindia.org/dispap/dis38.pdf
32. Sawhney M, Coutinho L. Dukh Dard and Gam: Living with chronic conditions of the painful kind. 2001. http://www.iegindia.org/dispap/dis29.pdf
33. Singh S, Prasad R, Verma R, Pandey A. Psychosocial, cultural and service factors affecting reproductive morbidity among rural women in Maharashtra (Summary report). 2001. http://www.iipsindia.org/pub/res/repro_morbidity.pdf
34. Sood A, Singh G, Kulkarni P, Thakur R, Jagdish. An exploratory study on use of computer assisted learning (CAL) for in-service health management training programmes at National Institute of Health and Family Welfare. 2001. http://www.nihfw.org/asp/ResearchStudies.asp?currentpage=3
35. Sugathan K, Mishra V, Retherford R. Promoting institutional deliveries in rural India: the role of antenatal-care services. 2001. http://pdf.usaid.gov/pdf_docs/PNACN953.pdf
36. Taylor Nelson Sofres Mode Pvt Ltd, Family Health International. The Maharashtra (India) condom market: Product quality and supply study. 2001. http://www.fhi.org/en/HIVAIDS/pub/survreports/condommarket.htm
37. Thorat S. Database on child labour in India: an assessment with respect to nature of data, period and uses. 2001. http://www-wds.worldbank.org/external/default/WDSContentServer/WDSP/IB/2008/05/26/000333038_20080526025231/Rendered/PDF/438600WP0Box321aBase1India01PUBLIC1.pdf
38. United Nations Environment Programme. The state of environment - India: 2001. 2001. <http://envfor.nic.in/soer/list.html>

**2002**

1. Arora N, Devi R, Mathew T, Lakshman M, Rewal S, Anand K, Ganguly K, Adhish V, Goswami K, Pandav C, Biswas M. Vitamin A and iron folic acid supplementation 2001-2002. 2002. http://www.ipen.org.in/images/stories/Vitamin%20A%20full%20Report.pdf
2. Arora N, Lakshman M, Goswami K, Rewal S, Adhish S, Ganguly K, Biswas M. Family health awareness campaign - Concurrent evaluation. 2002. http://www.ipen.org.in/images/stories/fhac%20full%20report.pdf
3. Gulati S, Sharma S. Fertility and RCH status in Uttaranchal and Uttar Pradesh: A district level analysis. 2002. http://www.iegindia.org/workpap/wp225.pdf
4. Gumber A. Health insurance for the informal sector: problems and prospects. 2002. http://202.54.104.236/intranet/eip/whorep/uploads/H/H-Insurance%201.25.pdf
5. Gupta I, Sankar D. Health of the elderly in India: A multivariate analysis. 2002. http://www.iegindia.org/dispap/dis46.pdf
6. Gupta I, Sankar D. Medical attention at death: Evidence from India. 2002. http://www.iegindia.org/dispap/dis45.pdf
7. Gupta I, Sankar D. Treatment-seeking behaviour and the willingness to pay for antiretroviral therapy of HIV positive patients in India. 2002. http://www.iegindia.org/dispap/dis58.pdf
8. Indian Council of Medical Research. Five-year consolidated report of the hospital based cancer registries 1994-1998: An assessment of the burden and care of cancer patients. 2002. http://icmr.nic.in/ncrp/HBCR%202003.pdf
9. Indian Council of Medical Research. Two-year report of the population based cancer registries: Incidence and distribution of cancer 1997-98. 2002. http://icmr.nic.in/ncrp/PBCR%201997-1998/Starting%20Pages.pdf
10. Indian Institute of Health Management Research, Institute for Development Alternatives, Institute for Development Communication, The Naz Foundation Trust, International Centre for Research on Women. Men, masculinity and domestic violence in India: Summary report of four studies. 2002. http://www.icrw.org/docs/DV_India_Report4_52002.pdf
11. International Centre for Research on Women. Women-initiated community level responses to domestic violence: Summary report of three studies. 2002. http://www.icrw.org/docs/DVIndia_Report5_702.pdf
12. Jain N, Gupta S, Singh L. Meeting reproductive and sexual health needs of adolescents: A strategic approach for Rajasthan, India. 2002. http://www.iipsindia.org/resreport_auth_rks_qci.htm
13. Johnston H. Abortion practice in India - A review of literature (Abortion Assessment Project - India).2002. http://www.cehat.org/go/uploads/AapIndia/work1.pdf
14. Kothari D. Population projections for Rajasthan and districts: 2002-2011. 2002. http://www.iihmr.org/Publicationp/Occa/OC-PAPR3.pdf
15. Krishnaji N, James K. Gender differentials in adult mortality in India - with notes on rural-urban contrasts. 2002. http://www.cess.ac.in/cesshome/gender_diffrentials%5Cgender_diffrentials_WP.pdf
16. Lalitha N. India's pharmaceutical industry in the WTO regime: A SWOT analysis. 2002. http://www.gidr.ac.in/gidr/pdf/WP-131.pdf
17. Nagendranath A, Chari P. Health insurance in India: The emerging paradigms. 2002. http://202.54.104.236/intranet/eip/whorep/uploads/H/H-Insurance%201.78.pdf
18. National Nutrition Monitoring Bureau. Diet and nutritional status of rural population. 2002. http://www.nnmbindia.org/NNMBREPORT2001-web.pdf
19. Peters D, Yazbeck A, Sharma R, Ramana G, Pritchett L, Wagstaff A. Better health systems for India's poor: findings, analysis, and options. 2002.<http://www-wds.worldbank.org/external/default/WDSContentServer/WDSP/IB/2002/05/30/000094946_02051604053640/Rendered/PDF/multi0page.pdf>
20. Planning Commission. Planning Commission 10th five-year plan (2002-2007). 2002. http://planningcommission.nic.in/plans/planrel/fiveyr/welcome.html
21. Pushpangadan K. Social returns from drinking water sanitation and hygiene education: A case study of two coastal villages in Kerala. 2002. http://www.cds.edu/download_files/333.pdf
22. Saigal S. Literature review on service delivery in India. 2002. http://www-wds.worldbank.org/external/default/WDSContentServer/WDSP/IB/2003/10/15/000160016_20031015113936/Rendered/PDF/269360Literature0review0India.pdf
23. Shariff A, Singh G. Determinants of maternal health care utilisation in India: Evidence from a recent household survey. 2002. http://202.54.104.236/intranet/eip/whorep/uploads/H/H-Paper%2023.pdf
24. South Asia Regional Office – World Bank. Implementation completion report - Cataract blindness control project, India. 2002. http://www-wds.worldbank.org/external/default/WDSContentServer/WDSP/IB/2003/01/17/000094946_03010904013884/Rendered/PDF/multi0page.pdf
25. South Asia Regional Office – World Bank. Implementation completion report - Family welfare (urban slums) project. 2002. http://www-wds.worldbank.org/external/default/WDSContentServer/WDSP/IB/2003/03/07/000090000_03012304060921/Rendered/PDF/multi0page.pdf
26. Tiwari V. A pilot study on functioning of MIS under RCH Programme in states of UP and Maharashtra (2001-2002). 2002. http://www.nihfw.org/asp/ResearchStudies.asp?currentpage=4
27. Tiwari V. Study on functioning of sex-clinics in old part of Delhi: An exploratory study health services (2000-2002). 2002. http://www.nihfw.org/asp/ResearchStudies.asp?currentpage=3
28. Vasudeva U, Sood A, Datta U, Dhar N, Singh G. Evaluation study of the distance education course conducted at NIHFW (PG Certificate Course in Health and Family Welfare Management & PG Certificate Course in Hospital Management) 2001-2002. 2002. http://www.nihfw.org/asp/ResearchStudies.asp?currentpage=3
29. World Bank. Indoor air pollution: Energy and health for the poor - An assessment of the Deepan Scheme in Andhra Pradesh. 2002. http://www-wds.worldbank.org/external/default/WDSContentServer/WDSP/IB/2004/05/18/000090341_20040518132118/Rendered/PDF/284370Indoor0air0no.06.pdf
30. World Vision, United States Agency for International Development. World Vision: third annual review report -- Ballia rural integrated child survival project, Uttar Pradesh, India. 2002. http://pdf.usaid.gov/pdf_docs/PDABU719.pdf

**2003**

1. Alam M, Mukherjee M. Ageing, ADL disabilities and need for public health initiatives. 2003. http://www.iegindia.org/workpap/wp241.pdf
2. Anand A. Sexuality, abortion and the media: A review of adolescent concerns (Abortion Assessment Project - India). 2003. http://www.cehat.org/go/uploads/AapIndia/work2.pdf
3. Batra S, Rabindranathan S. Abortion training in India: A long way to go (Abortion Assessment Project - India). 2003. http://www.cehat.org/go/uploads/AapIndia/work7.pdf
4. Bhalla S, Saigal S, Basu N.Girl's education is it - nothing else matters (much). 2003. http://www-wds.worldbank.org/external/default/WDSContentServer/WDSP/IB/2004/03/01/000265513_20040301102825/Rendered/PDF/28016.pdf
5. Bhat R, Babu S. Health insurance and third party administrators: Issues and challenges. 2003. http://202.54.104.236/intranet/eip/whorep/uploads/H/H-Insurance%201.99.pdf
6. Catalyst Consortium. Indian focus groups on birth spacing: qualitative study in India. 2003. http://pdf.usaid.gov/pdf_docs/PNADF207.pdf
7. Centre for Advocacy and Research, Positive Women's Network. Positive speaking: Voices of women living with HIV/AIDS. 2003. http://www.unifem.org.in/genderandhivaids.html
8. CEPDA/India, Krishi Gramin Vikas Kendra. Enabling the expansion & sustainability of integrated RCH [reproductive & child health]/infectious diseases outreach services in Ranchi, Jharkhand: final evaluation. 2003. http://pdf.usaid.gov/pdf_docs/PDABY764.pdf
9. Cornu C, Attawell K. Involvement of people living with HIV/AIDS in community-based prevention, care and support programs in developing countries: a multi-country diagnostic study. 2003. http://pdf.usaid.gov/pdf_docs/PNACW036.pdf
10. Das Gupta M, Khaleghian P, Sarwal R. Governance of communicable disease control services: a case study and lessons from India. 2003. http://www-wds.worldbank.org/external/default/WDSContentServer/WDSP/IB/2003/08/30/000094946_03082104020547/Rendered/PDF/multi0page.pdf
11. Das J, Sanchez-Paramo C. Short but not sweet - new evidence on short duration morbidities from India. 2003. http://www-wds.worldbank.org/external/default/WDSContentServer/WDSP/IB/2003/03/22/000094946_03030704153292/Rendered/PDF/multi0page.pdf
12. Dilip T, Duggal R. Demand for public health services in Mumbai. 2003. http://www.cehat.org/go/uploads/Keastward/dphcm.pdf
13. Energy & Infrastructure Unit, South Asia Region, World Bank. Implementation completion report - Uttar Pradesh and Uttaranchal rural water supply and environmental sanitation (Swajal) project. 2003.http://www-wds.worldbank.org/external/default/WDSContentServer/WDSP/IB/2003/11/25/000090341_20031125143759/Rendered/PDF/27288.pdf
14. Ghosh S. "Professional" abortion seekers: the sex-workers of Kolkata (Abortion Assessment Project - India). 2003. http://www.cehat.org/go/uploads/AapIndia/work9.pdf
15. Gupta I, Datta A. Inequities in health and health care in India - Can the poor hope for a respite? 2003. http://www.iegindia.org/dispap/dis80.pdf
16. Gupta I, Panda S, Motihar R. HIV/AIDS and development in India - Background study for the Swedish Country Strategy for India. 2003. http://www.sida.se/sida/jsp/sida.jsp?d=118&a=2642&language=en_US
17. Gupta S. Adolescent and youth reproductive health in India: status, issues, policies, and programs. 2003. http://pdf.usaid.gov/pdf_docs/PNACT789.pdf
18. Guruswamy M, Mohanty S. Projected users of condoms, oral pills, IUD and their estimated cost in India. 2003. http://www.iipsindia.org/resreport_auth_mg_iudopc.htm
19. Indian Council of Medical Research. Lymphatic filariasis: field studies & evaluation of control strategies. 2003. http://icmr.nic.in/000520/lymphatic_filariasis.pdf
20. Indian Council of Medical Research. Estimates of maternal mortality ratios in India and its states - a pilot study. 2003. http://icmr.nic.in/final/Final%20Pilot%20Report.pdf
21. Indian Institute of Health Management Research. Accessibility and utilisation of RNTCP services by SC/ST population. 2003. http://www.tbcindia.org/pdfs/Accessibility%20and%20Utilisation%20of%20RNTCP%20Services%20by%20SC-ST%20Population%20-%20IIHMR.pdf
22. International Institute of Population Sciences, International Centre for Research on Women. Realizing reproductive choice and rights: Abortion and contraception in India. 2003. http://www.icrw.org/docs/RCA_India_Report_0303.pdf
23. Jejeebhoy S, Sebastian M. Actions that protect: Promoting sexual and reproductive health and choice among young people in India. 2003. http://www.popcouncil.org/asia/indiaRI.html
24. Joint United Nations Development Programme, World Bank Energy Sector Management Assistance Programme. India: access of the poor to clean household fuels. 2003. http://www-wds.worldbank.org/external/default/WDSContentServer/WDSP/IB/2003/11/19/000012009_20031119134452/Rendered/PDF/2630030India.pdf
25. Joshi R. Perinatal and neonatal mortality in rural Punjab: A community based case-control study. 2003. http://www.sctimst.ac.in/amchss/publications/wp/wp_3.pdf
26. Kumar B. Target free approach for Family Welfare in Gujarat: A review of policy and its implications. 2003. http://www.gidr.ac.in/gidr/pdf/WP-143.pdf
27. Mallik R. 'Negative choice' - Sex determination and sex selective abortion in India (Abortion Assessment Project - India). 2003. http://www.cehat.org/go/uploads/AapIndia/work6.pdf
28. Mavalankar D. Policy barriers preventing access to emergency obstetric care in rural India. 2003. http://www.sctimst.ac.in/amchss/publications/wp/wp_5.pdf
29. Mavalankar D, Ramani K, Shaw J. Management of RH services in India and the need for health system reform. 2003. http://www.iimahd.ernet.in/publications/data/2003-09-04mavalankar.pdf
30. Mishra A, Levitt-Dayal M. Improving adolescent reproductive health knowledge and outcomes through NGO youth-friendly services. 2003. http://pdf.usaid.gov/pdf_docs/PNACU211.pdf
31. Mishra U, Dilip T. Assessing potential for induced abortion among Indian women (Abortion Assessment Project - India). 2003. http://www.cehat.org/go/uploads/AapIndia/work4.pdf
32. Murty M, Gulati S. Banerjee A. Health benefits from urban air pollution abatement in the Indian Subcontinent. 2003. http://www.iegindia.org/workpap/wp236.pdf
33. Nanda S, Ram F. Teenage motherhood, child survival and child health: Evidences from National Family Health Survey, India. 2003. http://www.gidr.ac.in/gidr/pdf/WP-134.pdf
34. National Nutrition Monitoring Bureau. Prevalence of micronutrient deficiencies. 2003. http://www.nnmbindia.org/NNMB%20MND%20REPORT%202004-Web.pdf
35. National Sample Survey Organisation. Report on village facilities - NSS 58th round (July-December 2002). 2003. http://mospi.nic.in/rept%20_%20pubn/ftest.asp?rept_id=487&type=NSSO
36. National Sample Survey Organisation. Disabled persons in India - NSS 58th round (July-December 2002). 2003. http://mospi.nic.in/rept%20_%20pubn/ftest.asp?rept_id=485&type=NSSO
37. National Sample Survey Organisation. Condition of urban slums: Salient Findings. NSS 58th Round (July 2002-December 2002). 2003. http://mospi.nic.in/rept%20_%20pubn/ftest.asp?rept_id=486&type=NSSO
38. Onursal B. Health care waste management in India: lessons from experience. 2003. http://www-wds.worldbank.org/external/default/WDSContentServer/WDSP/IB/2005/06/13/000090341_20050613121330/Rendered/PDF/325050PAPER0IN1r0official0use0only1.pdf
39. ORG Centre for Social Research. Baseline study on accessibility and utilisation of RNTCP services by PLWHA. 2003. http://www.tbcindia.org/pdfs/Baseline%20Study%20on%20Accessibility%20and%20Utlization%20of%20RNTCP%20Services%20by%20PLWHA%20-%20ORG%20CSR.pdf
40. ORG Centre for Social Research. Knowledge, attitude, behaviour and practices (KABP) survey of male reproductive and sexual health among truckers and cleaners/helpers in three cities of Jharkhand. 2003. http://pdf.usaid.gov/pdf_docs/PNACW632.pdf
41. Population Council. Introducing DMPA [depot medroxyprogesterone acetate] injectable contraceptives to private medical practitioners in urban Gujarat. 2003. http://pdf.usaid.gov/pdf_docs/PNACS379.pdf
42. Population Services International. The Balbir Pasha story: an innovative approach to reducing HIV/AIDS prevalence through targeted mass media communications in Mumbai, India. 2003.<http://pdf.usaid.gov/pdf_docs/PNADE789.pdf>
43. Q Market Research. Project Befikar: A management report for an attitudes and perceptions study among truckers in India. 2003. http://www.psi.org/research/fr/306%20India%20Truckers.pdf
44. R K Swamy. Baseline KAP study under RNTCP Project. 2003. http://www.tbcindia.org/pdfs/Baseline%20KAP%20Study%20under%20RNTCP%20Project%20-%20CMS.pdf
45. Ramachandran V. Social sector reforms in India - Background study for the Swedish Country Strategy for India 2003-2007. 2003. http://www.sida.se/sida/jsp/sida.jsp?d=118&a=2640&language=en_US
46. Ray C, Gupta P, de Beyer J. Research on tobacco in India (including the betel quid and areca nut): an annotated bibliography of research on use, health effects, economics and control efforts. 2003. http://www-wds.worldbank.org/external/default/WDSContentServer/WDSP/IB/2004/05/26/000265513_20040526152329/Rendered/PDF/288960Ray1Research0on1whole.pdf
47. Retherford R, Roy T. Factors affecting sex-selective abortion in India and 17 major states. 2003. http://pdf.usaid.gov/pdf_docs/PNACR987.pdf
48. Saha S. Methodological issues in abortion estimation (Abortion Assessment Project - India). 2003. http://www.cehat.org/go/uploads/AapIndia/work8.pdf
49. Santhya K. Changing the family planning scenario in India: An overview of recent evidence. 2003. http://www.popcouncil.org/pdfs/wp/seasia/seawp17.pdf
50. Schraman B, Sodani P. Final report: Expert mission on health financing / health insurance training needs and dialog programmes in India. 2003. http://202.54.104.236/intranet/eip/whorep/uploads/H/H-Paper%2022.pdf
51. Seshadri S, Subramaniyam P, Jha P. The potential demand for and strategic use of an HIV-1 vaccine in Southern Asia. 2003.<http://www-wds.worldbank.org/external/default/WDSContentServer/WDSP/IB/2003/06/30/000094946_03061204065029/Rendered/PDF/multi0page.pdf>
52. Sharma S. Adolescent fertility in selected states of India. 2003. http://www.iegindia.org/dispap/dis79.pdf
53. Singh S, Verma R, Prasad R. The impact of HIV/AIDS related deaths on households and their coping strategies: a study with special reference to children. 2003. http://www.iipsindia.org/pub/res/impact_of_hiv.pdf
54. South Asia Regional Office – World Bank. Implementation completion report - Second Integrated Child Development Services project, India. 2003. http://www-wds.worldbank.org/external/default/WDSContentServer/WDSP/IB/2003/04/30/000160016_20030430174534/Rendered/PDF/256101IN12nd0integrated0child0dev1ICR.pdf
55. Sundar R. Abortion costs and financing - A review (Abortion Assessment Project - India). 2003. http://www.cehat.org/go/uploads/AapIndia/work5.pdf
56. Swedish International Development Cooperation Agency. Performance analysis 2003 Part 1: General & sectoral analysis - India. 2003. http://www.sida.se/sida/jsp/sida.jsp?d=118&a=17681&language=en_US
57. Tamm G, Rao R, Urwitz V. Evolving strategies for better health and development of adolescent/young people. 2003.<http://www.sida.se/sida/jsp/sida.jsp?d=118&a=2593&language=en_US>
58. Taylor Nelson Sofres Mode Pvt Ltd. Feasibility survey report enabling the expansion and sustainability of integrated RCH [reproductive and child health]/infectious diseases outreach services through skill upgradation, capacity building and women's empowerment in Ranchi, Jharkhand. 2003. http://pdf.usaid.gov/pdf_docs/PNACU210.pdf
59. Unisa S, Prakasam C, Sinha R, Bhagat R. Evidence of sex selective abortion from two cultural settings of India: A study of Haryana and Tamil Nadu. 2003. http://www.iipsindia.org/resreport_auth_rks_ssa.htm
60. United States Agency for International Development. Involving men to address gender inequities. 2003. http://pdf.usaid.gov/pdf_docs/PNACU658.pdf
61. World Bank. Project performance assessment report - National AIDS control project, India. 2003. http://www-wds.worldbank.org/external/default/WDSContentServer/WDSP/IB/2003/08/30/000094946_03082104011041/Rendered/PDF/multi0page.pdf
62. World Health Organization India Country Office. Country report for Mode 1: Cross-border trade in health services - (E-health). India - Country level report. 2003. http://www.whoindia.org/EN/Section2/Section233_538.htm
63. Yazbeck A, Peters D (Editors). Health policy research in South Asia: building capacity for reform. 2003. http://www-wds.worldbank.org/external/default/WDSContentServer/WDSP/IB/2003/12/08/000090341_20031208111101/Rendered/PDF/272100PAPER0Health0policy0research.pdf

**2004**

1. Academy for Educational Development. AED/LINKAGES/India: final report (1997-2004). 2004. http://pdf.usaid.gov/pdf_docs/PDACD544.pdf
2. Administrative Staff College of India. Gender differentials in the Revised National Tuberculosis Control Programme. 2004. http://www.tbcindia.org/pdfs/Gender%20Differentials%20in%20the%20RNTCP%20-%20ASCI.pdf
3. Ahuja R. Health insurance for the poor in India. 2004. http://202.54.104.236/intranet/eip/whorep/uploads/H/H-Insurance%201.27.pdf
4. Annigeri V, Prosser L, Reynolds J, Roy R. Assessment of public-private partnership opportunities in India. 2004. http://pdf.usaid.gov/pdf_docs/PNADC694.pdf
5. Arora N, Devi S, Pandey R, Krishna V, Kumar H, Adhish S, Chaturvedi S, Biswas M, Chadha S, Uppal S. Injection practices in India. 2004. http://www.ipen.org.in/downloads/PDF/AIPI.pdf
6. Balakrishnan K, Mehta S, Kumar P, Ramaswamy P, Sambandam S, Kumar S, Smith K. Indoor air pollution associated with household fuel use in India: an exposure assessment and modelling exercise in rural districts of Andhra Pradesh. 2004. http://www-wds.worldbank.org/external/default/WDSContentServer/WDSP/IB/2007/01/12/000310607_20070112151617/Rendered/PDF/383490IN0Indoor0air0pollution01PUBLIC1.pdf
7. Barge S, Bracken H, Elul B, Kumar N, Khan W, Verman S, Camlin C. Formal and informal abortion services in Rajasthan, India: Results of a situation analysis. 2004. http://www.popcouncil.org/pdfs/abortion_analysis_india.pdf
8. Belli P, Heywood P, Anand N. West Bengal: health policy note. 2004. http://www-wds.worldbank.org/external/default/WDSContentServer/WDSP/IB/2005/01/19/000160016_20050119091038/Rendered/PDF/30296.pdf
9. Belli P, Lee Y, Heywood P, Pruthi H, Anand N, Khan M. Assam health policy note. 2004. http://www-wds.worldbank.org/external/default/main?pagePK=64193027&piPK=64187937&theSitePK=523679&menuPK=64187510&searchMenuPK=51634624&theSitePK=523679&entityID=000090341_20041028100437&searchMenuPK=51634624&theSitePK=523679
10. Bhat R, Jain N. Time series analysis of private healthcare expenditures GDP: Cointegration results with structural breaks. 2004. http://www.iimahd.ernet.in/publications/data/2004-05-10rbhat.pdf
11. Bhat R, Maheshwari S. Human resource issues and its implications for health sector reforms. 2004. http://www.iimahd.ernet.in/publications/data/2004-01-04rameshbhat.pdf
12. Bhat R, Maheshwari S, Saha S. Treating HIV/AIDS patients in India with antiretroviral therapy: a management challenge. 2004. http://www.iimahd.ernet.in/publications/data/2004-06-03rbhat.pdf
13. Bhat R, Saha S. Financing issues in proposed HIV/AIDS intervention of providing anti-retroviral drugs to selected regions in India. 2004. http://www.iimahd.ernet.in/publications/data/2004-05-01rbhat.pdf
14. Bhattacharya M. Involvement of Panchayati Raj Institutions in environmental health - an intervention study. 2004. http://www.nihfw.org/asp/ResearchStudies.asp?currentpage=3
15. Castle C. Expanding care and support in South India: scaling up YRG CARE's patient-centered approach. 2004. http://pdf.usaid.gov/pdf_docs/PNACY163.pdf
16. Catalyst Consortium. Perspectives on birth spacing in five countries: Bolivia, Egypt, India, Pakistan and Peru. 2004. http://pdf.usaid.gov/pdf_docs/PNADF210.pdf
17. Centre for Enquiry into Health and Allied Themes. Abortion Assessment Project - India: Research summaries and abstracts. 2004. http://www.cehat.org/go/uploads/AapIndia/summary.pdf
18. Costello-Daly C. Maximizing resources to meet client needs: evaluation of a comprehensive HIV/AIDS care and support model in India. 2004. http://pdf.usaid.gov/pdf_docs/PNACY165.pdf
19. Counterpart International. 'Gift of Life' (Jeevan Daan) child survival program: final evaluation report, August 9 to September 13, 2004. 2004. http://pdf.usaid.gov/pdf_docs/PDACD016.pdf
20. Das J, Hammer J. Which doctor? Combining vignettes and item response to measure doctor quality. 2004. http://www-wds.worldbank.org/external/default/WDSContentServer/WDSP/IB/2004/07/23/000112742_20040723160111/Rendered/PDF/wps3301.pdf
21. Das U, Nandan D. Qualitative assessment of the incorporation of the standard days method into a community-based program in Sitapur: a focus on male involvement. 2004. http://pdf.usaid.gov/pdf_docs/PNADB589.pdf
22. Datta U. Study on functioning of health worker female and male in India. 2004. http://www.nihfw.org/asp/ResearchStudies.asp?currentpage=2
23. Deolalikar A. India - Attaining the Millennium Development Goals in India: role of public policy and service delivery. 2004. http://www-wds.worldbank.org/external/default/WDSContentServer/WDSP/IB/2005/01/07/000090341_20050107091547/Rendered/PDF/302660IN.pdf
24. Dhar N. A study on identification of training needs and designing curriculum of NSS volunteers for creating community awareness on population stabilization. 2004. http://www.nihfw.org/asp/ResearchStudies.asp?currentpage=1
25. Duggal R, Barge S. Abortion services in India: Report of a multicentric enquiry (Abortion Assessment Project - India). 2004. http://www.cehat.org/go/uploads/AapIndia/national.pdf
26. Elul B, Barge S, Verman S, Kumar N, Bracken H, Sadhvani H. Unwanted pregnancy and induced abortion: Data from men and women in Rajasthan, India. 2004. https://www.popcouncil.org/pdfs/unwanted.pdf
27. Elul B, Bracken H, Verma S, Ved R, Singhi R, Lockwood K. Unwanted pregnancy and induced abortion in Rajasthan, India: A qualitative exploration. 2004. https://www.popcouncil.org/pdfs/SAReport.pdf
28. Foundation for Research in Health Systems. Community involvement in reproductive health: findings from research in Karnataka, India. 2004. http://pdf.usaid.gov/pdf_docs/PNADA527.pdf
29. Goyal S, Biswal P, Ranganathan K. Economic history of tobacco production in India. 2004. http://58.68.105.146/pdf/tobacco_1.PDF
30. Gupta I, Trivedi M. Coverage for antiretrovirals: Alternatives for care and support organisations. 2004. http://www.iegindia.org/dispap/dis93.pdf
31. Gupta I, Trivedi M. Social health insurance redefine: Health for all through coverage for all. 2004. http://www.iegindia.org/dispap/dis90.pdf
32. Hemanta M. Economic burden of HIV/AIDS: A study on HIV/AIDS patients. 2004. http://www.nihfw.org/asp/ResearchStudies.asp?currentpage=2
33. Hirve S. Abortion policy in India: Lacunae and future challenges (Abortion Assessment Project - India). 2004. http://www.cehat.org/go/uploads/AapIndia/hirve.pdf
34. Indian Council of Medical Research. Development of an atlas of cancer in India: First all India report 2001-2002. 2004. http://202.54.104.236/intranet/NMH/Can-atlas/Home.htm
35. Indian Council of Medical Research. Assessment of burden of non-communicable diseases. 2004. http://www.whoindia.org/LinkFiles/Assessment_of_Burden_of_NCD_Assessment_of_Burden_of_NCDs_Updated.pdf
36. Institute of Social Sciences, National Human Rights Commission, United Nations Development Fund for Women. A report on trafficking in women and children in India 2002-2003. 2004.<http://nhrch.nic.in/Publications/ReportOnTrafficking.pdf>
37. Kalaivani K. Operationalisation of risk identification and referral using antenatal card. 2004. http://www.nihfw.org/asp/ResearchStudies.asp?currentpage=3
38. Kapilashrami M. Operational research on community and facility based interventions for making pregnancy safer. 2004. http://www.nihfw.org/asp/ResearchStudies.asp?currentpage=2
39. Kaul V, Priyadarshi M, Heywood P, Singh S, Sipahimalani-Rao V, Sankar D, Ramana G. Reaching out to the child: an integrated approach to child development. 2004. http://www-wds.worldbank.org/external/default/WDSContentServer/WDSP/IB/2004/08/16/000160016_20040816101728/Rendered/PDF/29695.pdf
40. Khot A, Menon S, Dilip T. Domestic violence: levels, correlates, impact and response. A community based study of married women from Mumbai slums. 2004. http://www.cehat.org/go/uploads/AarogyachaMargavar/aarogyachamargavar.pdf
41. Kumar B. Primary health care in Gujarat: Evidence on Utilisation, mis-matches and wastage. 2004. http://www.gidr.ac.in/gidr/pdf/WP-149.pdf
42. Maheshwari S, Bhat R. Challenges in sustaining a hospital: lessons for managing healthcare institutions. 2004. http://www.iimahd.ernet.in/publications/data/2004-02-03sunilm.pdf
43. Marimuthu P. Morbidity study in the slums of Delhi - a pilot study. 2004. http://www.nihfw.org/asp/ResearchStudies.asp?currentpage=3
44. Ministry of Health and Family Welfare. Health sector reforms in India: Initiatives from 9 states. 2004. http://www.nhicindia.org/content/wrindia/Nupur/doc7011131200953.pdf
45. Ministry of Health and Family Welfare. India Country Report: Population and development - 10 years since ICPD. 2004. http://india.unfpa.org/?publications=365
46. Mittal P, Wijeyaratne P, Pandey S. Status of insecticide resistance of malaria, kala-azar and Japanese encephalitis vectors in Bangladesh, Bhutan, India and Nepal (BBIN). 2004. http://pdf.usaid.gov/pdf_docs/PNACX375.pdf
47. Nagarajan,G. Medical tourism in India: Strategy for its development. 2004. http://202.54.104.236/intranet/eip/whorep/uploads/H/H-MEDICAL%20TOURISM%20IN%20INDIA%20STRATEGY%20FOR%20ITS.pdf
48. Over M, Heywood P, Gold J, Gupta I, Hira S, Marseille E. HIV/AIDS treatment and prevention in India: modelling the cost and consequences. 2004. http://www-wds.worldbank.org/external/default/WDSContentServer/WDSP/IB/2004/09/21/000090341_20040921112225/Rendered/PDF/297060PAPER0HIV0Aids0India.pdf
49. Padma G. Maternal morbidity in rural Andhra Pradesh. 2004. http://www.cess.ac.in/cesshome/wp%5Cwp-63.pdf
50. Pai A, Atri R, Shiveshwarkar S, Sivadas A. An outreach intervention in Manipur, Injecting drug users and their sexual partners. 2004. http://www.sida.se/sida/jsp/sida.jsp?d=118&a=3206&language=en_US
51. Peters D, Rao K, Ramana G. India - equity effects of quality improvements on health services utilization and patient satisfaction in Uttar Pradesh state. 2004. http://www-wds.worldbank.org/external/default/WDSContentServer/WDSP/IB/2004/11/19/000090341_20041119135607/Rendered/PDF/304760RPP5IndiaUP.pdf
52. Population Council, CARE-India. Integrating adolescent livelihood activities within a reproductive health program for urban slum dwellers in India. 2004. http://pdf.usaid.gov/pdf_docs/PNADA283.pdf
53. Raju K, Rayappa P, Rao K, Sivakami M. Normative and actual provision of antenatal health care services in Karnataka. 2004. http://www.isec.ac.in/WP%20-%20145.pdf
54. Ramachandran T. Prevalence of overweight and obesity among school and college going adolescents in rural and urban Thiruvananthapuram districts, Kerala, India. 2004. http://www.sctimst.ac.in/amchss/publications/wp/wp_7.pdf
55. Ranson M, Joshi P, Shah M, Shaikh Y. India - assessing the reach of three SEWA health services among the poor. 2004. http://www-wds.worldbank.org/external/default/main?pagePK=64193027&piPK=64187937&theSitePK=523679&menuPK=64187510&searchMenuPK=64187295&theSitePK=523679&entityID=000090341_20041118101429&searchMenuPK=64187295&theSitePK=523679
56. Rao S. The WHO Global strategy on diet, physical activity and health: relevance and implications for India. 2004. http://www.whoindia.org/LinkFiles/Health_Promotion_HP_dr_sushila.pdf
57. Rehman I, Malhotra P (Editors). Fire without smoke: learning from the national program on improved Chulhas. 2004.<http://www-wds.worldbank.org/external/default/WDSContentServer/WDSP/IB/2006/12/11/000011823_20061211142432/Rendered/PDF/38124.pdf>
58. Singh S, Gupta K, Lahiri S, Schensul J. Prevention of HIV/AIDS among migrant youth in low-income slums of Mumbai (Summary Report). 2004. http://www.iipsindia.org/pub/res/prevention_hiv.pdf
59. Singh S, Lahiri S, Srivastava H, Roy T. A qualitative assessment of emergency obstetric care in 3 districts of Maharashtra: A border district cluster strategy for women's right to life and health (Summary report). 2004. http://www.iipsindia.org/pub/res/obstetric_care.pdf
60. Singh S, Verma R, Roy T. Expanded and informed contraceptive choice in Maharashtra: Assessing barriers to and opportunities for policy implementation. 2004. http://www.iipsindia.org/pub/res/contra_choice.pdf
61. South Asia Regional Office – World Bank. Implementation completion report - Second state health systems development project, India. 2004. http://www-wds.worldbank.org/external/default/WDSContentServer/WDSP/IB/2004/10/06/000090341_20041006094839/Rendered/PDF/300660IN.pdf
62. Swain P. Sociodemographic and health profile of widows in India. 2004. http://www.nihfw.org/asp/ResearchStudies.asp?currentpage=2
63. Taneja S, Agarwal S. Situational analysis for guiding USAID/India and EHP/India: technical assistance efforts in Indore, Madhya Pradesh, India. 2004. http://pdf.usaid.gov/pdf_docs/PNACY560.pdf
64. Tekhre Y. Assessment of contraceptive acceptance among males in district Kullu, Himachal Pradesh. 2004. http://www.nihfw.org/asp/ResearchStudies.asp?currentpage=2
65. Tiwari V. Review study on national health information system in two states of India. 2004. http://www.nihfw.org/asp/ResearchStudies.asp?currentpage=1
66. Tobgay K. Health seeking behaviour and delays in diagnosis and treatment in patients reporting with cough of three weeks or more to tuberculosis units and microscopy centres in East Sikkim. 2004. http://www.sctimst.ac.in/amchss/publications/wp/wp_6.pdf
67. Unisa S, Jagannath P, Shekhar C, Dhir V, Roy T. An epidemiological study to investigate the gallbladder diseases in North India. 2004. http://www.iipsindia.org/resreport_auth_su_gb.htm
68. United Nations Population Fund. Experiences of users and providers with progestin only injectable contraceptive - DMPA: Findings of a multi-centric study. 2004. http://india.unfpa.org/drive/ExpandingContraceptiveoptions.pdf
69. Varkey L, Mishr, A, Das A, Ottolenghi E, Huntington D, Adamchak S, Khan M. Involving men in maternity care in India. 2004. http://pdf.usaid.gov/pdf_docs/PNACX587.pdf
70. Varkey S. Community led initiatives for child survival program (CLICS): annual report – 67 villages in Wardha District, Maharashtra State, India: October 1, 2003 to September 30, 2004. 2004. http://pdf.usaid.gov/pdf_docs/PDACA727.pdf
71. Wijeyaratne P, Valecha N, Joshi A, Singh D, Pandey S. An inventory on malaria drug resistance in Bangladesh, Bhutan, India and Nepal. 2004. http://pdf.usaid.gov/pdf_docs/PNACY099.pdf
72. World Vision. First annual review: September 13th-17th, 2004 -- PRAGATI [protecting and advancing gains] child survival project. 2004. http://pdf.usaid.gov/pdf_docs/PNADA973.pdf
73. World Vision. Detailed implementation plan: PRAGATI [protecting and advancing gains] child survival project. 2004. http://pdf.usaid.gov/pdf_docs/PDACA790.pdf

**2005**

1. Academy of Nursing Studies. Situational analysis of public health nursing personnel in India: Based on national review and consultations in six states. 2005. http://www.whoindia.org/LinkFiles/HSD_Resources_Situation_Analysis_of_Public_Health_Nursing_Personnel.pdf
2. Administrative Staff College of India. Millennium Development Goals and health - India. 2005. http://www.whoindia.org/EN/Section2/Section400.htm
3. Administrative Staff College of India. Review of studies on financing of drug and pharmaceuticals in India - Report. 2005. http://www.whoindia.org/LinkFiles/Traditional_Medicine_Review_of_Studies_on_Financing_of_Drugs_and_Pharmaceuticals_.pdf
4. Ahuja R. Health insurance for the poor in India: An analytical study. 2005. http://202.54.104.236/intranet/eip/whorep/uploads/H/H-Insurance%201.26.pdf
5. Bhat R, Maheshwari S, Saha S. Third party administrators and health insurance in India: Perception of providers and policyholders. 2005.<http://www.iimahd.ernet.in/publications/data/2005-01-02.pdf>
6. Bhat R, Rajagopal S. Preliminary analysis of claims data to understand relationship between disease patterns and quality of care and its implications for health insurance in India. 2005. http://www.iimahd.ernet.in/publications/data/2005-09-03ramesh.pdf
7. Centre for Women's Studies. A situational analysis of domestic violence against women in Kerala. 2005. http://wcd.nic.in/research/Domestic%20Voilence-Kerala.pdf
8. Chakrabartty A. HIV counselling, testing and referral services in mental healthcare settings in Kolkata - A provider perspective. 2005. http://www.sctimst.ac.in/amchss/publications/WP/wp_9.pdf
9. Chakraborty S. Health seeking behaviour of aged population of a rural block in West Bengal. 2005. http://www.sctimst.ac.in/amchss/publications/wp/wp_8.pdf
10. Chittaranjan National Cancer Institute. Epidemiological study of air pollution related children's health in rural, suburban and urban areas of West Bengal. 2005. http://www.whoindia.org/EN/Section33/Section132_1578.htm
11. Das J, Hammer J. Money for nothing: the dire straits of medical practice in Delhi, India. 2005. http://www-wds.worldbank.org/external/default/WDSContentServer/WDSP/IB/2005/07/20/000016406_20050720164402/Rendered/PDF/wps3669.pdf
12. Das N, Shah U, Chitania V, Patel P, Khan M, Mishra A, Foreit J. Systematic screening to integrate reproductive health services in India. 2005.<http://pdf.usaid.gov/pdf_docs/PNADD881.pdf>
13. Davies J, Chan C, Vibha. Final evaluation: social marketing strategies for maternal and child health in the States of Uttar Pradesh, Uttaranchal & Jharkhand, India -- October 2002-May 2005. 2005. http://pdf.usaid.gov/pdf_docs/PDACF308.pdf
14. Department of Health and Family Welfare - Government of Gujarat, World Health Organization. Rapid assessment and action planning process (RAAPP) in Gujarat, India: A method and tools to enable Ministries of Education and Health to assess and strengthen their capacity to promote health through schools. 2005. http://www.whoindia.org/LinkFiles/Health_Promotion_Final_RAAPP_report-4august.pdf
15. Desai T. On the optimal staffing of surgeons and efficient scheduling of surgeries at a high-volume eye hospital. 2005. http://www.iimahd.ernet.in/publications/data/2006-04-08rbhat.pdf
16. DosajhU, Ghosh I, Lundren R. Feasibility of incorporating the standard days method into CASP family planning services in urban slums of India. 2005. http://pdf.usaid.gov/pdf_docs/PNADG768.pdf
17. Duggal R, Dilip T, Raymus P. Health and healthcare in Maharashtra. 2005. http://www.cehat.org/go/uploads/Hhr/hhcm.pdf
18. Federation of Indian Chambers of Commerce and Industry. Competitiveness of the Indian pharmaceutical industry in the new product patent regime. 2005. http://202.54.104.236/intranet/eip/whorep/uploads/H/H-COMPETITIVENESS%20OF%20THE%20INDIAN.pdf
19. Gangolli L, Duggal R, Shukla A. Review of healthcare in India. 2005. http://www.cehat.org/go/uploads/Hhr/rhci.pdf
20. Goyal R, Khanna A. Reproductive health of adolescents in Rajasthan: A situational analysis. 2005. http://www.iihmr.org/Publicationp/Workingp/6.pdf
21. Gulati S. Poverty, RCH-care utilisation and fertility in India: A district level analysis. 2005. http://www.iegindia.org/workpap/wp257.pdf
22. Gupta I, Trivedi M,. Voluntary insurance sector in India: Partnering to achieve greater coverage for health. 2005. http://www.iegindia.org/dispap/dis95.pdf
23. Gupta S, Dash P, Kanjilal B. Strengthening district health system through management interventions. 2005. http://www.iihmr.org/Publicationp/ResearchB/JUNE2005%20-%20MS.pdf
24. Gupta S, Khanna A. Causes of maternal mortality in Rajasthan: A community based study. 2005. http://www.iihmr.org/Publicationp/ResearchB/APR-2005%20-%20MS%20.pdf
25. Hajeebhoy N, Karmali A, Tandon T. Final report: Gujarat health system development project (October 1998-December 31, 2004). 2005. http://pdf.usaid.gov/pdf_docs/PDACF017.pdf
26. Indian Council of Medical Research. Mental health research in India (Technical monograph on ICMR mental health studies). 2005. http://icmr.nic.in/publ/Mental%20Helth%20.pdf
27. Indian Council of Medical Research. Consolidated report of hospital based cancer registry for the year 1999-2000. 2005. http://icmr.nic.in/ncrp/1999-00/hospital_based.htm
28. Indian Council of Medical Research. Two-year report of the population based cancer registries 1999-2000. 2005. http://icmr.nic.in/ncrp/1999-00/PBCR%20Report%201999_00.pdf
29. Indian Council of Medical Research. National environmental health profile and comparative health risk assessment - Bangalore city. 2005. http://www.whoindia.org/LinkFiles/Environmental_Epidemiology_NEHP_Report_Bangalore.pdf
30. Indian Council of Medical Research, Population Council. Studying the utilisation of emergency contraceptive services through paramedics in India. 2005. https://www.popcouncil.org/pdfs/frontiers/research_updates/RU5_ECP_India.pdf
31. Indian Institute of Health Management Research. Study on institutional assessment of RNTCP - Report. 2005. http://www.tbcindia.org/pdfs/Study%20on%20Institutional%20Assessment%20of%20RNTCP%20-%20IIHMR.pdf
32. International Institute of Population Sciences. India Facility Survey (under Reproductive and Child Health Project) Phase II, 2003. 2005. http://www.rchiips.org/PRCH-2.html
33. International Institute of Population Sciences, Johns Hopkins University. Quality of care in Indian Family Planning programme and reproductive behaviour of women in four Indian states: A follow-up study of NFHS-2. 2005. http://www.iipsindia.org/resreport_auth_rks_qci.htm
34. Karuna Trust. A healthy change: Community health insurance. 2005. http://data.undp.org.in/hiv/CHI.pdf
35. Khan M, Mishra A, Morankar S. Promoting role model of 'responsible man': gatekeepers' views on young men's sexual and reproductive health needs in Uttaranchal. 2005. http://pdf.usaid.gov/pdf_docs/PNADF310.pdf
36. Kumar D, Bhawsar R. Quality of care: Infrastructure, human resources and service utilisation - findings from concurrent evaluation of Reproductive and Child Health in Rajasthan, India. 2005. http://www.iihmr.org/Publicationp/Policy%20Briefs/POLICY%20-%20DHIRENDRA%20.pdf
37. Kumar S, Gupta S, Bhatt S, George A, Bhattacharya A. Men, masculinity and domestic violence against women. 2005. http://www.iihmr.org/Publicationp/ResearchB/JAN-2005%20-%20MS.pdf
38. Lalitha N. Essential drugs in Government healthcare: Emerging model of procurement and supply. 2005. http://www.gidr.ac.in/gidr/pdf/WP-161.pdf
39. Lamaute D, Jha A, Gupta A. India insurance sector assistance review. 2005. http://pdf.usaid.gov/pdf_docs/PNADG470.pdf
40. Maheshwari S, Bhat R, Saha S. Human resources practices and commitment of senior officials in health system: reflections from a progressive state in a developing economy. 2005. http://www.iimahd.ernet.in/publications/data/2005-09-02ramesh.pdf
41. Maheshwari S, Bhat R, Saha S. Directions for reforms in the health sector: reflections from a state in a developing country. 2005. http://www.iimahd.ernet.in/publications/data/2005-01-03.pdf
42. Mathivazhagan T. A pilot study on communication strategy for reaching the unreached tribal population in Mandla district of Madhya Pradesh. 2005. http://www.nihfw.org/asp/ResearchStudies.asp?currentpage=1
43. Ministry of Health and Family Welfare. National Health Accounts India 2001-2. 2005. http://www.whoindia.org/LinkFiles/NHA_India_NHA_2001-02.pdf
44. Ministry of Health and Family Welfare. Performance needs assessment of basic health care workers in immunisation in India. 2005. http://www.whoindia.org/LinkFiles/Routine_Immunization_Performance_Needs_Assessment_(PNA)_of_Basic_Health_Care_Workers_in_Immunization_in_India_(2005)_.pdf
45. Ministry of Health and Family Welfare, World Health Organization, Centre for Disease Control. Report on tobacco control in India. 2005.<http://www.whoindia.org/EN/Section20/Section25_516.htm>
46. Ministry of Statistics and Programme Implementation. Millennium Development Goals India Country Report 2005. 2005. http://mospi.nic.in/rept%20_%20pubn/ftest.asp?rept_id=ssd04_2005&type=NSSO
47. Mukherji S, Priyadarshi M, Singh S. Communication in public health programmes: the leprosy project in India. 2005. http://www-wds.worldbank.org/external/default/main?pagePK=64193027&piPK=64187937&theSitePK=523679&menuPK=64187510&searchMenuPK=64187295&theSitePK=523679&entityID=000090341_20050415153656&searchMenuPK=64187295&theSitePK=523679
48. Nanda S. Micro-determinants of human fertility: Study of selected physiological and behavioural variables in SC and ST population. 2005. http://www.gidr.ac.in/gidr/pdf/WP-152.pdf
49. National Commission on Macroeconomics and Health. Report of the National Commission on Macroeconomics and Health. 2005. http://mohfw.nic.in/reports/reports/Report_on_NCMH/ReportoftheNationalCommission.pdf
50. ORG Centre for Social Research. Social assessment study for RNTCP. 2005. http://www.tbcindia.org/pdfs/Social%20Assessment%20Study%20for%20RNTCP%20-%20Final%20Report%20-%20ORG%20CSR.pdf
51. Pathfinder International. Promoting change in reproductive behaviour of youth: Pathfinder International's PRACHAR Project, Bihar, India. 2005.<http://www.pathfind.org/site/DocServer/India-Prachar_Project.pdf>
52. R K Swamy. Midterm KAP evaluation study under RNTCP Project - 2005. 2005. http://www.tbcindia.org/pdfs/Mid%20Term%20KAP%20Evaluation%20Study%20under%20RNTCP%20Project%20-%202005%20-%20CMS.pdf
53. Ram F, Mohanty S. State of human development in states and districts of India. 2005. http://www.iipsindia.org/resreport_auth_fr_hd.htm
54. Ram F, Shekhar C, Mohanty S. Human development: Strengthening district level vital statistics in India. 2005. http://www.iipsindia.org/resreport_auth_cs_hd.htm
55. Ram U. Endline evaluation of the RTI sub-project in Nashik, Maharashtra. 2005. http://www.iipsindia.org/resreport_auth_ur_rti.htm
56. Ram U. ISM&H beneficiaries covered under CGHS & selected teaching hospitals attached to ISMH College Mumbai. 2005. http://www.iipsindia.org/resreport_auth_ur_ismh.htm
57. Ramani K, Mavalankar D. Health system in India: Opportunities and challenges for improvements. 2005. http://www.iimahd.ernet.in/publications/data/2005-07-03ramani.pdf
58. Ramani K, Mehadiratta S, Patel A, Joshi D, Patel N. Urban health status in Ahmedabad city: GIS based study of Baherampura, Kubernagar, and Vasna wards. 2005.<http://www.iimahd.ernet.in/publications/data/2005-03-05ramani.pdf>
59. Ross S, Mankad D, Haheebhoy N, Tomaro J. Final evaluation: Gujarat health system development project (GHSDP): Sidhpur and Junagadh, India . 2005. http://pdf.usaid.gov/pdf_docs/PDACF016.pdf
60. Sharma S. Child health and nutritional status of children: The role of sex differentials. 2005. http://www.iegindia.org/workpap/wp262.pdf
61. Shekhar C, Ram F. National report on evaluation of functioning of Urban Health Posts/Urban Family Centres in India. 2005.<http://www.iipsindia.org/resreport_auth_cs_nr.htm>
62. Singh S, Lhungdim H, Chattopadhyay A, Roy T. Women's vulnerability to STI/HIV in India - Finding of the CHARCA Baseline Study. 2005. http://www.iipsindia.org/pub/res/vulnerability.pdf
63. Society for Economic Development & Environmental Management. Nutritional status of women and children and working of ICDS in flood-prone districts of Bihar. 2005. http://wcd.nic.in/research/Nutritional_status_bihar.doc
64. Society for Economic Development & Environmental Management, New Delhi. Quick review of working of ICDS in Rajasthan. 2005. http://wcd.nic.in/research/quick_review_icds_raj.pdf
65. Sodani P. Willingness to join health insurance: Results from Rajasthan pilot study for informal sector. 2005. http://www.iihmr.org/Publicationp/Policy%20Briefs/POLICY%20-%20SODANI%20.pdf
66. Sodhi G. World Vision India-CATALYST/India partnership on 'PRAGATI' project report. 2005. http://pdf.usaid.gov/pdf_docs/PNADF225.pdf
67. South Asia Regional Office – World Bank. For a breath of fresh air: Ten years of progress and challenges in urban air quality management in India, 1993-2002. 2005.<http://www-wds.worldbank.org/external/default/WDSContentServer/WDSP/IB/2006/01/27/000160016_20060127124503/Rendered/PDF/350470PAPER0IN0Breath0of0fresh0air.pdf>
68. South Asia Regional Office – World Bank. Implementation completion report - Second national leprosy elimination project. 2005. http://www-wds.worldbank.org/external/default/WDSContentServer/WDSP/IB/2005/06/27/000160016_20050627121117/Rendered/PDF/32044.pdf
69. Sri Ramachandra Medical College and Research Institute. Baseline assessment of environmental health status in Chennai - Project report. 2005. http://www.whoindia.org/LinkFiles/Environmental_Epidemiology_Environmental_Health_Status_in_Chennai.pdf
70. Swaminathan P. Trapped into living: Women's work environment and their perceptions of health. 2005. http://www.cehat.org/go/uploads/Engender/engender.pdf
71. TATA Institute of Social Sciences. Causes of farmer suicides in Maharashtra: An enquiry. 2005. http://tiss.edu/Causes%20of%20Farmer%20Suicides%20in%20Maharashtra.pdf
72. United States Agency for International Development. USAID/India: strategic objective close-out report -- reduced fertility and improved reproductive health in North India: FY 1994-2002. 2005. http://pdf.usaid.gov/pdf_docs/PDACF358.pdf
73. United States Agency for International Development. USAID/India: strategic objective close-out report -- improved child survival and nutrition in selected areas of India: FY 1994-2002. 2005. http://pdf.usaid.gov/pdf_docs/PDACF359.pdf
74. United States Agency for International Development. USAID/India: strategic objective close-out report -- reduced transmission and mitigated impact of infectious diseases, especially STD/HIV/AIDS in India: FY 1994-2002. 2005. http://pdf.usaid.gov/pdf_docs/PDACF361.pdf
75. United States Agency for International Development. The USAID/India Urban Health Program: An evaluation of activities to date and recommendations for the future. 2005. http://pdf.usaid.gov/pdf_docs/PDACF849.pdf
76. World Health Organization India Country Office, All India Institute of Medical Sciences, CARE, Department for International Development UK. India universal immunisation programme review. 2005. http://www.whoindia.org/LinkFiles/Routine_Immunization_Acknowledgements_contents.pdf
77. RNTCP Environmental Assessment Study. 2005. http://www.tbcindia.org/pdfs/Env%20asses.%20Executive_sumary_9th_april.pdf

**2006**

1. AC Nielsen ORG-MARG. Social assessment of HIV/AIDS among tribal people in India - A report. 2006. http://www-wds.worldbank.org/external/default/WDSContentServer/WDSP/IB/2006/11/17/000020953_20061117141000/Rendered/PDF/IPP1780REVISED1eople0in0India0FINAL.pdf
2. Alagarajan M, Kulkarni P. Trends in religious differentials in fertility, Kerala, India: An analysis of birth intervals. 2006. http://www.gidr.ac.in/gidr/pdf/WP-167.pdf
3. Anderson M, Arora N, Bartlett A, Kumar R, Khanna R, Nath L, Haeften R. Reproductive and child health nutrition and HIV/AIDS program (RACHNA): final evaluation. 2006.<http://pdf.usaid.gov/pdf_docs/PDACI026.pdf>
4. Bhat R, Jain N. Factoring affecting the demand for health insurance in a micro-insurance scheme. 2006. http://www.iimahd.ernet.in/publications/data/2006-07-02rbhat.pdf
5. Bhat R, Jain N. Financial performance of private sector hospitals in India: Some further evidence. 2006. http://www.iimahd.ernet.in/publications/data/2006-04-08rbhat.pdf
6. Bhat R, Singh A, Maheshwari S, Saha S. Maternal health financing - Issues and options: A study of Chiranjeevi Yojana in Gujarat. 2006. http://www.iimahd.ernet.in/publications/data/2006-08-03rbhat_Revised_Mar07.pdf
7. Centre for Market Research & Social Development. Field impact study of NACO campaign. 2006. http://www.nacoonline.org/upload/Publication/IEC%20&%20Mainstreaming/Field%20Impact%20Study%20of%20NACO%20Campaign%20A%20Report.pdf
8. Centre for North East Studies and Policy Research. Final report on functioning of Anganwadi centres in Assam and Meghalaya. 2006. http://wcd.nic.in/research/ICDS-Assam-Meghalaya.doc
9. Chatterjee C. Identities in motion: Migration and health in India. 2006. http://www.cehat.org/go/uploads/Hhr/migrants.pdf
10. Chaudhuri,P. Sexual harassment in the workplace: Experiences of women in the health sector. 2006. http://www.popcouncil.org/pdfs/wp/India_HPIF/001.pdf
11. Chaurasia A. Fertility transition in India: 1985-2003. 2006. http://www.iegindia.org/workpap/wp278.pdf
12. Chaurasia A. Mortality transition in urban India 1971-2002. 2006. http://www.iegindia.org/dispap/dis113.pdf
13. Contractor Q, Madhiwalla N, Gopal M. Uprooted homes, uprooted lives: A study on the impact of involuntary resettlement on a slum community in Mumbai. 2006. http://www.cehat.org/go/uploads/InvoluntaryResettlement/uprootedreport.pdf
14. CUTS Centre for Competition, Investment & Economic Regulation. Options for using competition law/policy tools in dealing with anti-competitive practices in the pharmaceutical industry and the health delivery system. 2006. http://www.nhicindia.org/content/wrindia/Nupur/doc7011131200958.pdf
15. Dhar B, Gopakumar K. Post 2005 TRIPS scenario in patent protection in the pharmaceutical sector: The case of the generic pharmaceutical industry in India. 2006. http://202.54.104.236/intranet/eip/whorep/uploads/H/H-Post-2005%20TRIPS%20scenario%20in%20patent%20protection%20in%20the%20pharmaceutical%20sector%20The%20case%20of%20the%20generic%20pharmaceutical%20industry%20in%20India.pdf
16. Gulati S. Fertility, MCH-care and poverty in India: Simultaneous structural analysis. 2006. http://www.iegindia.org/dispap/dis110.pdf
17. Gulati S. Beyond National Rural Health Mission 2005: Issues of national concern. 2006.<http://www.iegindia.org/dispap/dis109.pdf>
18. Gupta I, Kandamuthan S, Upadhyaya D. Economic impact of cardiovascular diseases in India. 2006. http://www.iegindia.org/dispap/dis102.pdf
19. Gupta I, Skill C, Trivedi M, Rau A, Narang A, Mohan H. Covering treatment for HIV and AIDS in India: A feasibility study. 2006. http://data.undp.org.in/hiv/UNDPFeasabilityStudy.pdf
20. Gupta I, Trivedi M, Kandamuthn S. An analysis of recurrent costs of the free ART programme of the Government of India. 2006. http://www-wds.worldbank.org/external/default/WDSContentServer/WDSP/IB/2006/11/28/000020953_20061128122150/Rendered/PDF/380380IN0Costing0of0ART0Program01PUBLIC1.pdf
21. Gururaj G. Road traffic injury prevention in India. 2006. http://www.whoindia.org/LinkFiles/NMH_Resources_Road_Traffic_Injury_Prevention_in_India_Full_Report.pdf
22. Hammer J, Aiyar Y, Samji S. Bottom's up: to the role of Panchayati Raj Institutions in health and health services. 2006. http://www-wds.worldbank.org/external/default/WDSContentServer/WDSP/IB/2006/08/11/000310607_20060811135719/Rendered/PDF/369641SDP9801web.pdf
23. IC Health. National cardiovascular disease database. 2006. http://www.whoindia.org/LinkFiles/NMH_Resources_National_CVD_database-Final_Report.pdf
24. Indian Council of Medical Research. Consolidated report of population based cancer registries 2001-2004: Incidence and distribution of cancer. 2006. http://icmr.nic.in/ncrp/report_pop_2001-04/cancer_p_based.htm
25. Indian Council of Medical Research. Population based cancer registries under North Eastern regional cancer registry – First report: 2003-2004 – Incidence and distribution of cancer. 2006. http://icmr.nic.in/ncrp/first_report_2003-04/first_report.htm
26. Indian Council of Medical Research. Sentinel Health Monitoring Centres in India: Biochemical risk factor survey for non-communicable diseases. 2006. http://www.nhicindia.org/site/Search/HicsReports.asp?page=3
27. Indian Council of Medical Research. Database for disease burden estimation malaria, filaria, dengue and diarrhoeal diseases. 2006. http://www.whoindia.org/EN/Section2/Section427_1348.htm
28. International Institute of Population Sciences. Reproductive and child health district level household survey 2002-2004. 2006. http://www.rchiips.org/pdf/rch2/National_Report_RCH-II.pdf
29. International Institute of Population Sciences. Nutritional status of children and prevalence of anaemia among children, adolescent girls and pregnant women - District Level Household Survey on Reproductive and Child Health - India 2002-2004. 2006. http://www.rchiips.org/pdf/rch2/National_Nutrition_Report_RCH-II.pdf
30. International Institute of Population Sciences. Health system performance assessment: World Health Survey - India, 2003. 2006. http://www.whoindia.org/LinkFiles/WHS-India_Health_System_Performance_Assessment_Book.pdf
31. Kapur, A. The Impact of HIV/AIDS on women care givers in situations of poverty: Policy Issues. 2006. http://www.unifem.org.in/violenceagainstwomen.html
32. Kathuria V, Khan N. Environmental equity and vulnerability to air pollution: Evidence from Delhi, India. 2006. http://www.iegindia.org/workpap/wp269.pdf
33. KEM Hospital Research Centre, Population Council. Formation of partnerships among young women and men in Pune district, Maharashtra. 2006. https://www.popcouncil.org/pdfs/IndiaUpdate/IndiaUpdate_Pune.pdf
34. Krishna M. Protecting children for a healthy tomorrow: Lessons from the Andhra Pradesh Partnership Project on immunisation. 2006. http://www.path.org/files/CP_protecting_children.pdf
35. Kumar A, Jeyalakshmi S, Mukhopadhyay P, Gupta P. Improving and strengthening the use of ICD 10 and medical record system in India - A case study (2004 & 2005) - Report and recommendations. 2006.<http://www.cbhidghs.nic.in/writereaddata/mainlinkfile/Combined10.pdf>
36. Lalitha N, Joseph D. Patents and biopharmaceuticals in India: Emerging issues. 2006. http://www.gidr.ac.in/gidr/pdf/WP-168.pdf
37. Madras Medical College. Integrated response of health care systems to rapid population ageing (INTRA) - INTRA II. 2006. http://www.whoindia.org/LinkFiles/Health_Care_for_the_Elderly_NMH_INTRA_II.pdf
38. Madras Medical College. Integrated response of health care systems to rapid population ageing (INTRA) - INTRA III. 2006. http://www.whoindia.org/LinkFiles/Health_Care_for_the_Elderly_NMH_INTRA_III.pdf
39. Mahendra V, Gilborn L, George B, Samson L, Mudoi R, Jadav S, Gupta I, Bharat S, Daly C. Reducing AIDS-related stigma and discrimination in Indian hospitals. 2006. http://pdf.usaid.gov/pdf_docs/PNADG545.pdf
40. Mahendra V, Verma R, Pelto P, Pradhan S, Singh V, Rao A, Vij T. Context and dynamics of same-sex behaviour among long-distance truckers in India: Findings from qualitative research. 2006. http://www.popcouncil.org/pdfs/MSMIndiaResearchBrief.pdf
41. Maheshwari S, Bhat R, Saha S. Commitment of state health officials: Identifying factors and scope for improvement. 2006. http://www.iimahd.ernet.in/publications/data/2006-01-02sunil.pdf
42. Mani S. The sectoral system of innovation of Indian pharmaceutical industry. 2006. http://cds.edu/download_files/wp382.pdf
43. Ministry of Women and Child Development, Formative Research and Development Services. Kishori Shakti Yojana (KSY) under the ambit of ICDS in UP and Rajasthan. 2006. http://wcd.nic.in/research/ksyreport/executivesummary-ksy.pdf
44. Mishra M. Gendered vulnerabilities: Women's health and access to healthcare in India. 2006. http://www.cehat.org/go/uploads/Hhr/whahc.pdf
45. Mishra R. Dynamics of caste-based deprivation in child undernutrition in India. 2006. http://cds.edu/download_files/wp380.pdf
46. Narayana K. The unqualified medical practitioners: Methods of practice and nexus with the qualified doctors. 2006. [http://www.cess.ac.in/cesshome/wp%5Cwp-70.pdf](http://www.cess.ac.in/cesshome/wp\wp-70.pdf)
47. National Nutrition Monitoring Bureau. Diet and nutritional status of population and prevalence of hypertension among adults in rural areas. 2006. http://www.nnmbindia.org/NNMBReport06Nov20.pdf
48. National Nutrition Monitoring Bureau. Prevalence of vitamin A deficiency among preschool children in rural areas. 2006. http://www.nnmbindia.org/VAD-REPORT-final-21Feb07.pdf
49. National Program for Control of Blindness in India. Rapid assessment of trachoma in India: A report. 2006. http://www.nhicindia.org/content/wrindia/Nupur/doc7011201200979.pdf
50. National Sample Survey Organisation. Morbidity, health care and condition of the aged - NSS 60th round (January - June 2004). 2006. http://mospi.nic.in/rept%20_%20pubn/ftest.asp?rept_id=507&type=NSSO
51. Norwegian Agency for Development Cooperation. The Methodist Rural Public Health Programme - Evaluation. 2006. http://norad.no/en/Tools+and+publications/Publications/Publication+Page?key=117451
52. Nutrition Foundation of India. Report of evaluation of National Programme for Adolescent Girls Part I, II & III. 2006. http://wcd.nic.in/npagreport/NPAG1.pdf
53. Ojha V, Pradhan B. The macro-economic and sectoral impacts of HIV and AIDS in India: ACGE Analysis. 2006. http://www.ncaer.org/downloads/Reports/CGE.pdf
54. Pande R, Malhotra A. Son preference and daughter neglect in India: What happens to living girls? 2006. http://catalog.icrw.org/dbtw-wpd/exec/dbtwpub.dll
55. Pande R, Kurz K, Walia S, MacQuarrie K, Jain S. Improving the reproductive health of married and unmarried youth in India: Evidence of effectiveness and costs from community-based interventions, Final report of the adolescent reproductive health program in India. 2006. http://www.rockfound.org/about_us/news/2006/102306rep_india.shtml
56. Pandit Govind Ballabh Pant Institute of Studies in Rural Development. A study of children dependent on prostitutes in selected areas of Uttar Pradesh. 2006. http://wcd.nic.in/research/S.P.%20Pandey,%20Lucknow.doc
57. Population Council, CARE. Integrating adolescent livelihood activities within a reproductive health programme for urban slum dwellers in India. 2006. http://www.popcouncil.org/pdfs/IndiaUpdate/IndiaUpdate_Allahabad.pdf
58. Population Services International. India: HIV/AIDS TRaC Study among truck drivers, labourers, and fishermen in twelve port cities. 2006. http://www.psi.org/research/catalog_new.asp
59. Pradhan B, Sundar R. Gender: Impact of HIV and AIDS in India. 2006. http://www.ncaer.org/downloads/Reports/gender.pdf
60. Pradhan B, Sundar R, Singh S. Socioeconomic impact of HIV and AIDS in India. 2006. http://www.ncaer.org/downloads/Reports/India%20Report.pdf
61. Program for Appropriate Technology in Health. Strengthening immunisation services through service delivery support: The Andhra Pradesh experience. 2006.<http://www.path.org/files/CP_ap_experience.pdf>
62. R K Swamy. Endline evaluation study under RNTCP. 2006. http://www.tbcindia.org/pdfs/End%20Line%20KAP%20Study.pdf
63. Rachna W. Reproductive health practices and health seeking behaviour of female sex workers in Tamil Nadu. 2006. http://www.sctimst.ac.in/amchss/publications/WP/wp_12.pdf
64. Rajan S. Population ageing and health in India. 2006. http://www.cehat.org/go/uploads/Hhr/ageing.pdf
65. Ram F, Shekhar C. Ranking & mapping of districts based on socioeconomic and demographic indicators. 2006. http://www.iipsindia.org/resreport_auth_rks_qci.htm
66. Ram F, Sinha R, Mohanty S, Das A, Lakhani A, Haberland N, Santhya K. Marriage and motherhood: An exploratory study of the social and reproductive health status of married young women in Gujarat and West Bengal, India. 2006. http://www.popcouncil.org/pdfs/MarriageMotherhood.pdf
67. Ramani K, Mavalankar D, Patel A, Mahandiratta S, Bhardwaj R, Joshi D. A public-private partnership model for managing urban health: A study of Ahmedabad city. 2006. http://www.iimahd.ernet.in/publications/data/2006-03-05Ramani.pdf
68. Ranjan A. Obstetric risk and obstetric care in Central India. 2006. http://www.iegindia.org/dispap/dis105.pdf
69. Samuels F, Verma R, George C. Reducing HIV risk behaviours among key populations by increasing community involvement and building social capital: Baseline findings from Andhra Pradesh, India. 2006.<http://pdf.usaid.gov/pdf_docs/PNADG956.pdf>
70. Sarna A, Gupta I, Pujari S, Sengar A, Garg R, Weiss E. Examining adherence and sexual behaviours among patients on antiretroviral therapy in India. 2006.<http://pdf.usaid.gov/pdf_docs/PNADG546.pdf>
71. Saronjini N, Chakraborty S, Venkatachalam S, Bhattacharya S, Kapilashrami A, De R. Women's right to health. 2006.<http://nhrc.nic.in/Publications/Womens.pdf>
72. Save the Children. Abuse among child domestic workers: A research study in West Bengal. 2006. http://www.savethechildren.in/india/key_work/key_reports.html
73. Singh P. Relevance of trained traditional birth attendants in maternal health: Case study of Tehri Garhwal district, Uttaranchal state. 2006. http://www.sctimst.ac.in/amchss/publications/WP/wp_11.pdf
74. Sinha D. Tobacco control in schools in India (India Global Youth Tobacco Survey and Global School Personnel Survey, 2006). 2006. http://mohfw.nic.in/India%20Global%20Youth%20Tobacco%20Survey%20&%20Global%20School%20Personnel%20.pdf
75. Sinha D, Singh G. Tobacco control in medical schools of India (India Global Health Professional Student Survey, 2006). 2006. http://mohfw.nic.in/India%20Global%20Health%20Professional%20Student%20Survey,%202006.pdf
76. Society for Development Studies. Evaluation study of sub-mission (quality) projects under Accelerated Rural Water Supply Programmes - Final Report. 2006.<http://rural.nic.in/Eval_study_SMP_ARWSP.pdf>
77. South Asia Regional Office – World Bank. Implementation completion and results report - Woman and child development project, India. 2006. http://www-wds.worldbank.org/external/default/WDSContentServer/WDSP/IB/2006/12/18/000020953_20061218113012/Rendered/PDF/ICR000030.pdf
78. South Asia Regional Office – World Bank. Implementation completion and results report - Second National HIV/AIDS Control Project, India. 2006. http://www-wds.worldbank.org/external/default/WDSContentServer/WDSP/IB/2006/10/10/000112742_20061010101924/Rendered/PDF/ICR220REVISED.pdf
79. South Asia Regional Office – World Bank. Implementation completion and results report - Orissa health systems development project. 2006. http://www-wds.worldbank.org/external/default/WDSContentServer/WDSP/IB/2006/12/27/000020953_20061227102518/Rendered/PDF/icr12.pdf
80. South Asia Regional Office – World Bank. Implementation completion report - Maharashtra health systems development project. 2006. http://www-wds.worldbank.org/external/default/WDSContentServer/WDSP/IB/2006/06/07/000090341_20060607101630/Rendered/PDF/35390.pdf
81. TATA Institute of Social Sciences. Report of a rapid enquiry into the demolition and fire on the 9th May 2006 at Indiranagar and Jantanagar in Mandala, Mankhurd. 2006. http://tiss.edu/Report123.pdf
82. Taylor Nelson Mode India Pvt Ltd, Population Services International. HIV/AIDS Trac Study evaluating consistent condom use among male clients of female commercial sex workers in Andhra Pradesh, Karnataka, Maharashtra and Tamil Nadu. 2006. http://www.psi.org/research/catalog_new.asp
83. Thankappan K, Mini G. Surveillance of CVD risk factors and health promotion intervention in a rural community in Trivandrum district. 2006. http://www.whoindia.org/LinkFiles/NMH_Resources_CVD_prevention_tvm.pdf
84. Tiwari V, Nair K, Dhingra R, Shivdasani J, Kumar P . Evaluation of implementation status of National Iodine Deficiency Disorders Control Programme in India. 2006.<http://www.nihfw.org/html/Reports.htm>
85. United Nations Development Programme. A needs assessment study: People living with HIV/AIDS in Gujarat. 2006. http://data.undp.org.in/hiv/GSACS_NAIL.pdf
86. University of Kashmir, Tata Institute of Social Sciences, ActionAid International. The Jammu and Kashmir Earthquake: Damage and needs assessment report. 2006. http://tiss.edu/EarthQuake_2.pdf AND http://tiss.edu/EarthQuake_1.pdf
87. Varatharajan D, Anandan D. Re-activating primary health centres through industrial partnership in Tamil Nadu: Is it a sustainable model of partnership? 2006. http://www.sctimst.ac.in/amchss/publications/WP/wp_10.pdf
88. Virmani A. Poverty and hunger in India: What is needed to eliminate them. 2006.<http://planningcommission.nic.in/reports/wrkpapers/wk_pov106.pdf>
89. Visaria L, Barua A, Mistry R. Medical abortion: Some exploratory findings from Gujarat. 2006. http://www.gidr.ac.in/gidr/pdf/WP-166.pdf
90. Wilder J, Masilamani R, Mathew A. Reproductive health of young adults in India: The road to public health. Pathfinder International’s RHEYA Project demonstrates widespread community results in youth reproductive health. 2006. http://www.pathfind.org/site/DocServer/Pathfinder_Rheya.pdf?docID=7401
91. World Bank. Implementation completion report - Immunization strengthening project, India. 2006. http://www-wds.worldbank.org/external/default/WDSContentServer/WDSP/IB/2006/07/31/000090341_20060731101710/Rendered/PDF/35391.pdf

**2007**

1. Advent Healthcare Group. Human resources for pharmacy sector in India. 2007. http://www.whoindia.org/LinkFiles/Human_Resources_human_resources_for_pharmacy_sector.pdf
2. Ahuja R, Bhattacharya D. Healthy workplace in corporate sector - India: An operational research. 2007. http://www.nhicindia.org/content/wrindia/Nupur/doc7011191200967.pdf
3. Alam M. Is caring for elders an act of altruism? Some evidence from a household survey in Delhi. 2007. http://www.iegindia.org/dispap/dis121.pdf
4. Allison C, Ajwad I, Berman P, Das M, Krishna S, Das S, Kumar P. India - Achieving the millennium development goals (MDG) in India's poor states: reducing child mortality in Orissa. 2007. http://www-wds.worldbank.org/external/default/WDSContentServer/WDSP/IB/2007/06/26/000020439_20070626133557/Rendered/PDF/398550IN.pdf
5. Anand and Anand Advocates. Report on implications of the proposed Article 31bis of the TRIPS Agreement. 2007. http://www.nhicindia.org/content/wrindia/Nupur/doc7011131200957.pdf
6. Bapuji Dental College and Hospital. Oral health promotion and intervention activities carried out in rural areas of Davangere district. 2007. http://www.whoindia.org/LinkFiles/Oral_Health_ART_Project_Davangere.pdf
7. Bhat R, Chandra P, Mukherjee S. Involving private healthcare providers to reduce maternal mortality in India: A simulation study to understand implications on provider incentives. 2007. http://www.iimahd.ernet.in/publications/data/2007-01-01_SMukherjee.pdf
8. Bhat R, Jain N. A study of factors affecting renewal of health insurance policy. 2007. http://www.iimahd.ernet.in/publications/data/2007-01-02_rbhat.pdf
9. Bhat R, Maheshwari S, Saha S. Contracting-out reproductive and child health services through Mother NGO Scheme in India: Experiences and implications. 2007. http://www.iimahd.ernet.in/publications/data/2007-01-05_rbhat.pdf
10. Bhat R, Mavalankar D, Maheshwari S, Saha S. Provision of reproductive health services to urban poor through public-private partnerships: The case of Andhra Pradesh Urban Health Care Project. 2007. http://www.iimahd.ernet.in/publications/data/2007-01-07_rbhat.pdf
11. Bhat R, Mavalanker D, Singh P, Singh N. Maternal health financing in Gujarat: Preliminary results from a household survey of beneficiaries under Chiranjeevi Scheme. 2007.<http://www.iimahd.ernet.in/publications/data/2007-10-06Bhat.pdf>
12. Bora R. Imbalance in child sex ratio: Trends, causes and emerging issues. 2007. http://www.iegindia.org/workpap/wp280.pdf
13. Capps J, Canchi-Bhoopal K, Sawez N. Child survival Jeevan Daan: CS-XX expanded impact project, October 1, 2004-September 30, 2009: midterm evaluation report. 2007.<http://pdf.usaid.gov/pdf_docs/PDACL234.pdf>
14. CARE. Women's empowerment for better health outcomes. 2007. http://careindia.org/ManagePublications/VisitPublicationDetail.aspx?SectionID=118
15. Central Bureau of Health Intelligence. Managing human resources for health in India - A case study of Madhya Pradesh & Gujarat. 2007. http://www.cbhidghs.nic.in/writereaddata/mainlinkfile/File984.pdf
16. Central Pollution Control Board. Ground water quality status - Ground water quality series. 2007. http://www.cpcb.nic.in/oldwebsite/Water/ground_water.html
17. Centre for Peace and Development. Community needs assessment on HIV/AIDS in Mizoram. 2007. http://www.nacoonline.org/upload/Publication/M&E%20Surveillance,%20Research/COMMUNITY%20NEEDS%20ASSESSMENT%20ON%20HIV-AIDS%20IN%20MIZORAM.pdf
18. Chanda R. Foreign investment in hospitals in India: Status and implications. 2007. http://www.nhicindia.org/content/wrindia/Nupur/doc7011131200955.pdf
19. Chatrapati Shajui Maharaj Medical University. Promotion of oral health care and providing ART in two rural centres around Lucknow. 2007. http://www.whoindia.org/LinkFiles/Oral_Health_ART_Project_Lucknowmain_.pdf
20. Chatterjee C, Sheoran G. Vulnerable groups in India. 2007. http://www.cehat.org/go/uploads/Hhr/vulnerable.pdf
21. Chatterjee M, Levine R, Rao-Seshadri S, Murthy N. Better reproductive health for poor women in South Asia. 2007.<http://www-wds.worldbank.org/external/default/WDSContentServer/WDSP/IB/2008/01/08/000020953_20080108094613/Rendered/PDF/42027.pdf>
22. Chaurasia A. The age and sex structure of Tribal population in Central India. 2007. http://www.iegindia.org/dispap/dis117.pdf
23. Chittaranjan National Cancer Institute. Health effects of chronic exposure to smoke from biomass fuel burning in rural areas - Final report. 2007. http://www.whoindia.org/LinkFiles/Environmental_Epidemiology_biomass_fuel_burning_in_rural_areas.pdf
24. Das R, Biswas K, Panda P, Khan M, Homan R. Strengthening financial sustainability through integration of voluntary counselling and testing services with other reproductive health services. 2007. http://pdf.usaid.gov/pdf_docs/PNADI577.pdf
25. Deodhar S, Mahadiratta S, Ramani K, Mavalankar D, Ghosh S, Braganza V. Midday meal scheme: Understanding critical issues with reference to Ahmedabad city. 2007. http://www.iimahd.ernet.in/publications/data/2007-03-03_SatishDeodhar.pdf
26. Dr B Borooah Cancer Institute. Prevalence of precancerous lesions of the oral cavity and their association with areca nut and non-tobacco pan masala conforming areca nut in North Eastern states - A study. 2007.<http://www.whoindia.org/LinkFiles/Health_&_Environment_Health_areca_nut.pdf>
27. Dutta I, Bawari S. Health and healthcare in Assam. 2007. http://www.cehat.org/go/uploads/Hhr/assamreport1.pdf
28. Family Health International. India final report: October 1997-September 2007 -- USAID's implementing AIDS prevention and care (IMPACT) project. 2007. http://pdf.usaid.gov/pdf_docs/PDACK584.pdf
29. Federation of Indian Chamber of Commerce and Industry. FICCI study on implementation of food safety and standards act: An industry perspective. 2007. http://202.54.104.236/intranet/eip/whorep/uploads/H/H-FICCI%20study%20on%20Implementation%20of%20Food%20Safety%20and%20Standards%20Act.pdf
30. Government Dental College & Hospital. Management of dental caries in selected rural areas of Gujarat through atraumatic restorative technique - report. 2007. http://www.whoindia.org/LinkFiles/Oral_Health_ART_Project_ahmedabad.pdf
31. Gulati S, Chaurasia A, Singh R. Women's reproductive morbidity and treatment seeking behaviour in India. 2007. http://www.iegindia.org/dispap/dis120.pdf
32. Gulati S, Chaurasia A, Singh R. Unmet and met need of contraception in India. 2007.<http://www.iegindia.org/dispap/dis115.pdf>
33. Gupta I, Guin P. Health status and access to health services: A study of four slums. 2007. http://www.iegindia.org/dispap/dis122.pdf
34. IC Health. Cardiovascular disease (CVD) surveillance and health promotion in industrial settings: A module for CVD surveillance and health promotion. 2007. http://www.whoindia.org/LinkFiles/Non-communicable_Diseases_and_Mental_Health_NCD_risk_CVD_surveillance_for_industrial_settings.pdf
35. Indian Council of Medical Research. Consolidated report on hospital based cancer registries 2001-2003: An assessment of the burden and care of cancer patients. 2007. http://icmr.nic.in/ncrp/report_pop_hos_2001-03/cancer_h_based.htm
36. Indian Council of Medical Research, Population Council. Provision of emergency contraceptive services through paraprofessionals in India. 2007. http://pdf.usaid.gov/pdf_docs/PNADN559.pdf
37. Indian Pharmaceutical Association. Accreditation of pharmacies in India: A novel concept in community pharmacy in India. 2007. http://www.nhicindia.org/content/wrindia/Nupur/doc7011121200914.pdf
38. Institute of Health Systems. National AIDS Control Program, India: Institutional Assessment. 2007. http://www.nacoonline.org/upload/Finance/Institutional%20Assessment%20of%20National%20AIDS%20Control%20Programme.pdf
39. International Institute of Population Sciences, Macro International. National Family Health Survey (NFHS-3) India 2005-06 Volumes 1&2. 2007. http://www.nfhsindia.org/nfhs3_national_report.html
40. Johns Hopkins University, Indian Institute of Health Management Research, Indian Institute of Management. Draft report on Independent Evaluation of National AIDS Control Programme. 2007. http://www.nacoonline.org/upload/Finance/Draft%20Report%20on%20Independent%20Evaluation%20of%20NACP.pdf
41. Joseph J, Centre for Youth Development and Activities. Reflections on the campaign against sex selection and exploring ways forward: A study report. 2007. http://india.unfpa.org/drive/Reflections.pdf
42. Kacker L, Varadan S, Kumar P. Study on child abuse India. 2007. http://wcd.nic.in/
43. Kashyap R, Saha A, Rao M, Parikh D, Kulkarni P, Sinha S, Saiyed H. National environmental health profile and comparative health risk assessment - Ahmedabad. 2007. http://www.whoindia.org/LinkFiles/Environmental_Epidemiology_NEHP_Report_Ahmedabad.pdf
44. Kulkarni P. Estimation of missing girls at birth and juvenile ages in India. 2007. http://india.unfpa.org/?reports=379
45. Kumar A, Raut D, Gupta P, Singh U. Mortality statistics in India 2006 - Status of mortality statistics reporting in India. 2007. http://www.cbhidghs.nic.in/writereaddata/mainlinkfile/File976.pdf
46. Lakshmana C. Demographic change and gender inequality: A comparative study of Madhya Pradesh and Karnataka. 2007. http://www.isec.ac.in/WP%20-%20183.pdf
47. Lalitha N. Government intervention and prices of medicines: Lessons from Tamil Nadu. 2007. http://www.gidr.ac.in/gidr/pdf/WP-175.pdf
48. Lawyers' Collective Women's Rights Initiative. Staying Alive: First monitoring and evaluation report 2007 on the Protection of Women from Domestic Violence Act 2005. 2007. http://www.unifem.org.in/violenceagainstwomen.html
49. Lazmaiah A, Balakrishna N, Kumar S, Ranidranath M, Brahman G, Sesikeran B. Prevalence and determinants of overweight and obesity among urban adolescent school children, Andhra Pradesh India. 2007. http://www.whoindia.org/EN/Section20/Section385_1504.htm
50. Maharashtra State Pharmacy Council's Drug Information Centre. A study of misbranding and SALA drugs responsible for medication errors in Maharashtra and Gujarat. 2007. http://www.whoindia.org/LinkFiles/Essential_Drugs_Study_of_Misbranding_and_SALA_Drugs_Responsible_for_Medication_Errors_in_Maharashtra_&_Gujara.pdf
51. Mahatma Gandhi Postgraduate Institute of Dental Sciences. Atraumatic restorative treatment to the rural population. 2007.<http://www.whoindia.org/LinkFiles/Oral_Health_Atraumatic_Restorative_Treatment_to_the_Rural_Population.pdf>
52. Mahendra V, Mehrotra S, Srikanthi B, Panda S, Sarna A, Jayajsree A, Prasad R, Rutenberg N. Identifying areas for linkages between HIV and SRH for vulnerable populations: An exploratory study to assess female sex workers' sexual and reproductive health needs. 2007. http://www.popcouncil.org/pdfs/IndiaUpdate/IndiaUpdate_Linkages.pdf
53. Mahendra V, Mudoi R, Oinam A, Pakkela V, Sarna A, Panda S, Rau A, Singh L, Rutenberg N. Continuum of care for HIV-positive women accessing programs to prevent parent-to-child transmission : findings from India. 2007. http://pdf.usaid.gov/pdf_docs/PNADK418.pdf
54. Mahendra V, Pradha S, Swain S, Nanda A, Patnayak S, Panda S, Prasad R. Intergenerational communication on sexuality and HIV/AIDS: Exploring feasibility of building effective youth-adult partnerships to reduce HIV vulnerabilities of the young. 2007. http://www.popcouncil.org/pdfs/IndiaUpdate/IndiaUpdate_Intergenerational.pdf
55. Maulana Azad Dental College and Hospital. Oral heath status in rural child population - Promotional and interventional strategies. 2007.<http://www.whoindia.org/LinkFiles/Oral_Health_Oral_Health_Status_in_rural_child_population.pdf>
56. Mavalankar D, Kranti V, Bharati S. Strengthening midwifery services in India based on lessons from Sweden and Sri Lanka. 2007. http://www.iimahd.ernet.in/publications/data/2007-06-07Mavalankar.pdf
57. Mavalankar D, Shastri P, Jeram P, Ramani K. Chikungunya fever: A killer epidemic in Ahmedabad city. 2007. http://www.iimahd.ernet.in/publications/data/2007-06-02Mavalankar.pdf
58. Mavalankar D, Shastri P, Ramani K. Chikungunya epidemic mortality in India: Lessons from 17th century bills of mortality still relevant. 2007. http://www.iimahd.ernet.in/publications/data/2007-07-12Mavalankar.pdf
59. Mills A, Bos E, Lule E, Ramana G, Bulatao R. Obstetric care in poor settings in Ghana, India, and Kenya. 2007. http://www-wds.worldbank.org/external/default/WDSContentServer/WDSP/IB/2008/01/23/000310607_20080123112201/Rendered/PDF/418730REPLACEM1stetricCare01PUBLIC1.pdf
60. Ministry of Health and Family Welfare. Select health parameters: A comparative analysis across the National Sample Survey Organisation (NSSO) 42nd, 52nd and 60th Rounds. 2007. http://www.nhicindia.org/content/wrindia/Nupur/doc7011131200943.pdf
61. Ministry of Health and Family Welfare. Health sector reforms in India: Initiatives from states II. 2007. http://www.whoindia.org/EN/Section2/Section238_1257.htm
62. Ministry of Health and Family Welfare, World Health Organization. Oral health in India: A report of the multi centric study. 2007. http://whoindia.org/EN/Section20/Section30_1525.htm
63. Ministry of Health and Family Welfare, World Health Organization. Multicentric study to establish epidemiological data on health problems in elderly. 2007. http://www.whoindia.org/LinkFiles/Health_Care_for_the_Elderly_Multicentric_study_healthcareelderly_first.pdf
64. Mishra R. Nutritional deprivation among Indian pre-school children: Does rural-urban disparity matter? 2007. http://www.gidr.ac.in/gidr/pdf/WP-178.pdf
65. Misra, V, Ramasankar, P, Furga, L, Murty, J, Agarwal, S, Shah, P. Andhra Pradesh, India: improving health services through community score cards. 2007. http://www-wds.worldbank.org/external/default/WDSContentServer/WDSP/IB/2007/11/21/000020953_20071121132540/Rendered/PDF/415050India0Im1biltiy0no0101PUBLIC1.pdf
66. Mohapatra S, Gambhir I. Community based geriatric health care in Varanasi District - Final Report. 2007. http://www.whoindia.org/LinkFiles/Health_Care_for_the_Elderly_community_varanasi.pdf
67. Murty J, Agarwal S, Shah P, Kumar S. Maharashtra, India: Improving Panchayat service delivery through community score cards. 2007. http://www-wds.worldbank.org/external/default/WDSContentServer/WDSP/IB/2007/11/21/000020953_20071121135935/Rendered/PDF/415080IN0Case41August0200701PUBLIC1.pdf
68. Nair, K, Menon, V. Distress debt and suicides among agrarian households: Findings from three village studies in Kerala. 2007. http://cds.edu/download_files/wp397.pdf
69. Nandan D, Bhattacharya M. Annual HIV sentinel surveillance country report 2006. 2007. http://www.nacoonline.org/upload/NACO%20PDF/HIV%20Sentinel%20Surveillance%202006_India%20Country%20Report.pdf
70. Nandan D, Tiwari V, Kumar P. Study on functioning of Health & Family Welfare Societies in Haryana - Report. 2007. http://www.nihfw.org/html/Reports.htm
71. National Sample Survey Organisation. Nutritional intake in India 2004-2005 - NSS 61st round (July-June 2005).2007. http://mospi.nic.in/rept%20_%20pubn/ftest.asp?rept_id=513&type=NSSO
72. New Concept Information System. IEC - Baseline document Central TB Division. 2007. http://www.tbcindia.org/pdfs/IEC%20Baseline%20Document%20-%20August%202007.pdf
73. O'Keefe P. People with disabilities in India: from commitments to outcomes. 2007. http://www-wds.worldbank.org/external/default/WDSContentServer/WDSP/IB/2007/11/21/000310607_20071121124147/Rendered/PDF/415850IN0Disab1ort0NOV200701PUBLIC1.pdf
74. Population Council. Injecting drug users in India: Understanding sexual behaviours and sexual networks to design effective behaviour change strategies. 2007. http://www.popcouncil.org/pdfs/IndiaUpdate/IndiaUpdate_DrugUsers.pdf
75. Postgraduate Institute of Medical Education & Research. A community based integration program for control of non-communicable diseases in Union Territory of Chandigarh (Chandigarh Health Heart Action Project-CHHAP). 2007. http://www.whoindia.org/LinkFiles/NMH_Resources_chaap_report.pdf
76. Pradhan J. New policy regime and small pharmaceutical firms in India. 2007. http://202.54.104.236/intranet/eip/whorep/uploads/H/H-NEW%20POLICY%20REGIME%20AND%20SMALL%20PHARMACEUTICAL%20FIRMS%20IN%20INDIA.pdf
77. Premkumar R. Self-care activities in the biggest leprosy colony in India: Care after cure project in Prem Nagar, Champa, Chattisgarh state. 2007. http://www.whoindia.org/LinkFiles/Diability,_Injury_Prevention_&__Rehabilitation_disability_leprosy.pdf
78. Program for Appropriate Technology in Health. Options and challenges for converging HIV and sexual and reproductive health services in India: Findings from an assessment in Andhra Pradesh, Bihar, Maharashtra, and Uttar Pradesh. 2007. http://www.path.org/files/CP_India_cnvg_rpt.pdf
79. Rajalakshmi. Informed consent in sterilisation services: Evidence form public and private health care institutions in Chennai. 2007. https://www.popcouncil.org/pdfs/wp/India_HPIF/004.pdf
80. Rajalakshmi. Provider perspectives on informed consent in female sterilisation services: Finding from a facility-based study in Chennai. 2007. http://www.popcouncil.org/pdfs/wp/India_HPIF/003.pdf
81. Santhay K, Jejeebhoy S. Young people's sexual and reproductive health in India: Policies, programmes and realities. 2007. https://www.popcouncil.org/pdfs/wp/seasia/seawp19.pdf
82. Santhya K, Jejeebhoy S, Ghosh S, Haberland N. Addressing the sexual and reproductive health needs of young people: Perspectives and experiences of stakeholders from the health and non-health sector. 2007. http://www.popcouncil.org/pdfs/IndiaUpdate/IndiaUpdate_SRHYoungPeople.pdf
83. Sarna A, Ahmad J, Alexander G, Mahendra V, Rau A, Singh A, Rutenberg N. Exploring the barriers to accessing care and treatment for HIV-infected children in India: a diagnostic study. 2007. http://www.popcouncil.org/pdfs/IndiaUpdate/IndiaUpdate_Barriers.pdf
84. Schizophrenia Research Foundation. Report on the psycho social support programme in the tsunami affected areas of Cuddalore District in Tamil Nadu, India. 2007. http://www.nhicindia.org/content/wrindia/Nupur/doc7011201200985.pdf
85. Sharma S. Immunisation coverage in India. 2007. http://www.iegindia.org/workpap/wp283.pdf
86. Society for Economic and Social Studies. Public health safeguards in the Indian Patents Act and review of mailbox applications. 2007. http://www.nhicindia.org/content/wrindia/Nupur/doc7011131200959.pdf
87. South Asia Regional Office – World Bank. India - rural governments and service delivery (volumes 1 to 3). 2007. http://www-wds.worldbank.org/external/default/WDSContentServer/WDSP/IB/2007/03/20/000020439_20070320102407/Rendered/PDF/389011IN0v01.pdf
88. Srinivasan V, Masilamani R, Wilder J. Improved access to safe abortion care, Karnataka, India. 2007. http://www.pathfind.org/site/DocServer/Karnataka_IASAC_MAY07.pdf?docID=9061
89. Srivastava Y, Gopakumar K. Consumer drug information in India: A situational analysis. 2007. http://www.whoindia.org/LinkFiles/Traditional_Medicine_Consumer_Drug_Information_in_India%E2%80%93_A_Situational_Analysis.pdf
90. Sulabh International Academy of Environmental Sanitation. Final report: Study on disease burden due to inadequate water and sanitation facilities in India. 2007. http://www.whoindia.org/LinkFiles/Water_Quality_Disease_Burden_due_to_Inadequate_Water_&_Sanitation_Facilities_in_India.pdf
91. Tamm G, Rao R. Young people's health and development a reproductive and sexual health centred approach. 2007. http://www.sida.se/sida/jsp/sida.jsp?d=118&a=31491&language=en_US
92. Tiwari V. Study on functioning of health & family welfare societies in Haryana. 2007. http://www.nihfw.org/asp/ResearchStudies.asp?currentpage=1
93. United National Population Fund. Coordinated responses to violence against women: A documentation of interventions. 2007. http://india.unfpa.org/?publications=371
94. United Nations Development Programme. Migration gone wrong: Linkages between trafficking and HIV. 2007. http://data.undp.org.in/hiv/MigrationGoneWrong.pdf
95. United Nations Development Programme. Settled to move: The decision to migrate and its associated risks. 2007. http://data.undp.org.in/hiv/Settled2move.pdf
96. United Nations Development Programme. Push to open: An appraisal of sex workers' access to basic services. 2007. http://data.undp.org.in/hiv/Push2Open.pdf
97. United Nations Development Programme. Journey to safety: Information and communication technologies for reducing HIV and other vulnerabilities of migrants, their families and communities. 2007. http://data.undp.org.in/hivreport/UNDP%20ICT%20-%20Journey%20to%20Safety.pdf
98. Verma R, Saggurti N, Das M, RamaRao S, Jain A. Patterns and implications of male migration for HIV prevention strategies in Andhra Pradesh. 2007. http://www.popcouncil.org/pdfs/India_TechBrief.pdf
99. Virmani A. The sudoku of growth, poverty and malnutrition - Policy implications for lagging states. 2007. http://planningcommission.nic.in/reports/wrkpapers/wp07StJl12.pdf
100. World Bank. India - Country strategy progress report: for the period FY2005-2008.2007. http://www-wds.worldbank.org/external/default/WDSContentServer/WDSP/IB/2007/05/23/000020439_20070523093700/Rendered/PDF/39796.pdf
101. World Health Organization India Country Office. Not enough here... Too many there… Health workforce in India. 2007. http://www.whoindia.org/LinkFiles/Human_Resources_Health_Workforce_in_India_-_Apr07.pdf
102. World Health Organization India Country Office. A report on challenges and opportunities for pharmacists in health care in India.2007. http://www.whoindia.org/LinkFiles/Essential_Drugs_and_Medicines_Report_on_CO_for_Pharmacists_in_HC_in_India.pdf
103. Chronic disease detection and prevention programme for Malviya Nagar residents.2007. http://www.whoindia.org/LinkFiles/NMH_Resources_cvd_prevention_malviya.pdf
104. Management of acute coronary syndromes (ACS) in secondary care setting in Kerala, India: Impact of a quality improvement programme. 2007. http://www.whoindia.org/LinkFiles/NMH_Resources_cvd_MGMT__ace_report1.pdf
105. Report - New paradigm of medical care for persons with disabilities. 2007. http://www.whoindia.org/LinkFiles/Diability,_Injury_Prevention_&__Rehabilitation_disability_paradigm_tash.pdf
106. Surveillance of mortality and cardiovascular (CVD) related morbidity in industrial settings. 2007.<http://www.whoindia.org/LinkFiles/NMH_Resources_burden_cvd__mortality.pdf>

**2008**

1. Acharya A. Access and utilisation of health care services in urban low-income settlements in Surat, India. 2008. [http://www.css.ac.in/Working%20Paper%20-%205.pdf](http://www.css.ac.in/Working Paper - 5.pdf)
2. Agrawal S. Determinants of induced abortion and its consequences on women's reproductive health: findings from India's national family health surveys. 2008.<http://pdf.usaid.gov/pdf_docs/PNADM566.pdf>
3. Alam M. Ageing, socio-economic disparities and health outcomes: some evidence from rural India. 2008. http://www.iegindia.org/workpap/wp290.pdf
4. Alam M. Population ageing in South Asia: An overview and emerging issues of poverty and old age health. 2008. http://www.iegindia.org/dispap/dis123.pdf
5. Bedi J, Ramachandran H. Human development index for rural Andhra Pradesh. 2008. http://www.ncaer.org/Downloads/WorkingPapers/WP99.pdf
6. Chakraborty G, Nair A. State public health budget analysis Version 2.1. 2008. http://nhsrcindia.org/index.php?option=com_docman&task=doc_view&gid=63&tmpl=component&format=raw&Itemid=115
7. Coalition for Sustainable Nutrition Security in India. Overcoming the curse of malnutrition in India: a leadership agenda for action. 2008.<http://pdf.usaid.gov/pdf_docs/PNADM628.pdf>
8. Das J, Hammer J, Leonard K. The quality of medical advice in low-income countries. 2008. http://www-wds.worldbank.org/external/default/WDSContentServer/WDSP/IB/2008/01/30/000158349_20080130160825/Rendered/PDF/wps4501.pdf
9. Das J, Do Q, Friedman J, McKenzie D. Mental health patterns and consequences: results from survey data in five developing countries. 2008. http://www-wds.worldbank.org/external/default/WDSContentServer/WDSP/IB/2008/02/05/000158349_20080205131301/Rendered/PDF/wps4495.pdf
10. Dilip T. Role of private hospitals in Kerala: An exploration. 2008. http://cds.edu/admin/homeFiles/wp400.pdf
11. Gupta I, Trivedi M, Guin P. Understanding HIV and development: An analysis from Bellary districts in Karnataka, India. 2008. http://data.undp.org.in/hivreport/Bellary%20Report%20-%20English.pdf
12. Gupta P, Asma S. Bidi smoking and public health. 2008. [http://www.mohfw.nic.in/Bidi%20Smoking%20and%20Public%20Health.pdf](http://www.mohfw.nic.in/Bidi Smoking and Public Health.pdf)
13. Hemanta M. Utilisation of selected reproductive and child health services: Role of infrastructure facility in Uttar Pradesh, Punjab and Himachal Pradesh. 2008. http://www.nihfw.org/asp/ResearchStudies.asp?currentpage=1
14. Indian Council of Medical Research. Development of a feasibility module for road traffic injury surveillance. 2008. http://icmr.nic.in/final/final_report_traffic.pdf
15. Indian Council of Medical Research. North East population based cancer registries - Second report 2005-2006: Incidence and distribution of cancer. 2008.<http://www.pbcrindia.org/map1.htm>
16. Indian Council of Medical Research, National AIDS Control Organisation. Technical report: India HIV Estimates 2006. 2008. http://www.nacoonline.org/upload/NACO%20PDF/Technical%20Report%20on%20HIV%20Estimation%202006.pdf
17. Institute of Public Health. Training needs assessment for district health managers. 2008. [http://www.iphindia.org/new/images/Publications/srtt%20report.pdf](http://www.iphindia.org/new/images/Publications/srtt report.pdf)
18. International Institute of Population Sciences, Population Council. Sexuality education for youth in Maharashtra: yes or no? 2008. http://www.popcouncil.org/asia/indiaRI.html
19. John M, Kaur R, Palriwala R, Raju S, Sagar A. Planning families, planning gender: The adverse child sex ratio in selected districts of Madhya Pradesh, Rajasthan, Himachal Pradesh, Haryana and Punjab. 2008. http://www.cwds.ac.in/PlanningFamiliesPlanningGender.pdf
20. Johns Hopkins University, Indian Council of Medical Research. India Hib vaccine probe study Part A - Final report. 2008. http://pdf.usaid.gov/pdf_docs/PNADM961.pdf
21. Kanesathasan A, Cardinal L, Pearson E, Das Gupta S, Mukherjee S, Malotra A. Improving youth sexual and reproductive health through Disha, an integrated program in India. 2008. http://catalog.icrw.org/dbtw-wpd/exec/dbtwpub.dll
22. Karnataka Health Promotion Trust, Population Council. Migration/mobility and vulnerability to HIV among male migrant workers, Karnataka 2008-8. 2008.<http://www.popcouncil.org/asia/indiaRI.html>
23. Khan M, Mishra A, Sharma V, Varkey L. Development of a quality assurance procedure for reproductive health services for district public health systems: Implementation and scale-up. 2008. http://pdf.usaid.gov/pdf_docs/PNADL822.pdf
24. Kousalya P, Ganju D. Exploring positive women's lives in Namakkal district, India. 2008. http://www.popcouncil.org/pdfs/wp/India_HPIF/007.pdf
25. Kumar P, Simi T. Barriers to movement of health professionals: A case study of India. 2008.<http://www.whoindia.org/LinkFiles/Trade_Agreement_Barriers_to_Movement_of_Healthcare_Professionals.pdf>
26. Lawyers' Collective Women's Rights Initiative. Staying Alive: Second monitoring and evaluation report 2008 on the Protection of Women from Domestic Violence Act 2005. 2008. http://www.unifem.org.in/violenceagainstwomen.html
27. Malhotra S, Khot A, Nandraj S. Primary health care - Indian scenario. 2008. http://www.whoindia.org/LinkFiles/Health_Systems_Development_Primary_Health_Care.pdf
28. Mathiyazhagan T. Application of 'communication intervention package' and its effect on the behavioural patterns of tribal towards health care programmes in Mandla District of Madhya Pradesh. 2008. http://www.nihfw.org/asp/ResearchStudies.asp?currentpage=1
29. Mavalankar D, Kranti V. The changing role of auxiliary nurse midwife (ANM) in India: Implications for maternal and child health. 2008. http://www.iimahd.ernet.in/publications/data/2008-03-01Mavalankar.pdf
30. Ministry of Health and Family Welfare. Review of performance under Family Welfare Programmes during April 2008 - August 2008. 2008. http://mohfw.nic.in/Review%20of%20performance%20under%20FW%20programme%20during%20%20April-August%2008_PDF.xlsx.pdf
31. Ministry of Health and Family Welfare, World Health Organization. National Cancer Control Programme: Task Force Reports for XIth Plan. 2008.<http://www.whoindia.org/EN/Section20/Section385/Section401_1573.htm>
32. Nagarajan G, Murthy P. Mental health care and human rights. 2008.<http://nhrc.nic.in/Publications/Mental _Health _Care_and Human_Rights.pdf>
33. National AIDS Control Organisation. HIV sentinel surveillance and HIV estimation in India 2007 - a technical brief. 2008. http://www.nacoonline.org/upload/Publication/M&E%20Surveillance,%20Research/HIV%20Sentinel%20Surveillance%20and%20HIV%20Estimation%202007_A%20Technical%20Brief.pdf
34. National AIDS Control Organisation. Mainstreaming HIV and AIDS for women's empowerment. 2008. http://www.nacoonline.org/upload/Publication/IEC%20&%20Mainstreaming/mainstreaming%20hiv%20and%20aids%20for%20women.pdf
35. National Crime Records Bureau. Accidental deaths and suicides in India 2007. 2008. http://ncrb.nic.in/ADSI2007/home.htm
36. National Institute of Health and Family Welfare. Report of SIHFWs/CTIs for NRHM training. 2008. http://www.nihfw.org/html/Reports.htm
37. National Institute of Health and Family Welfare. Assessment and strengthening of training infrastructure available in the states and districts. 2008. http://www.nihfw.org/html/Reports.htm
38. National Sample Survey Organisation. Household expenditure in India, 2005-06; NSS 62nd Round (July 2005-June 2006). 2008. http://mospi.nic.in/rept%20_%20pubn/ftest.asp?rept_id=523&type=NSSO
39. ORG Centre for Social Research. National Behavioural Surveillance Survey (BSS) 2006 - Youth (15-24 years). 2008. http://www.nacoonline.org/upload/M&E%20Resources/Youth_report.pdf
40. Planning Commission. Planning Commission 11th five-year plan (2007-2012). 2008. http://planningcommission.nic.in/plans/planrel/fiveyr/welcome.html
41. Population Council. Patterns of migration/mobility and HIV risk among female sex workers, Andhra Pradesh, 2007-08. 2008. http://www.popcouncil.org/pdfs/India_FSWHIVAndhra.pdf
42. Population Council. Migration/mobility and vulnerability to HIV among male migrant workers, Andhra Pradesh 2007-08. 2008. https://www.popcouncil.org/pdfs/India_MaleMigrantHIVAndhra.pdf
43. Population Council, Department of Health and Family Welfare - Government of Gujarat, Centre for Operations Research & Training. Increasing the accessibility, acceptability and use of IUD in Gujarat, India. 2008. http://pdf.usaid.gov/pdf_docs/PNADN563.pdf
44. Population Council, Lala Lajpat Rai Memorial Medical College, Jamia Milia Islamia University. Promoting healthy timing and spacing of births in India through a community-based approach. 2008. http://pdf.usaid.gov/pdf_docs/PNADN566.pdf
45. Public Health Foundation of India, World Bank. India's health workforce: Size, composition and distribution. 2008. http://hrhindia.org/assets/images/Paper-I.pdf
46. Ram F, Ram U, Mohanty S, Singh A, Jejeebhoy S, Santhya K, Acharya R. Youth in India: Situation and needs 2006-2007, Maharashtra. 2008. http://www.iipsindia.org/gats/Report%20-%20Youth%20in%20India%20Situation%20and%20Needs.pdf
47. Ramani K, Mavalankar D, Tirupati D, Chand,V. Managerial challenges in addressing HIV/AIDS: Gujarat State AIDS Control Society (GSACS). 2008. http://www.iimahd.ernet.in/publications/data/2008-03-06Ramani.pdf
48. Rao M, Choudhury M. Inter-state equalisation of health expenditures in Indian Union. 2008. http://www.whoindia.org/LinkFiles/Health_Finance_Inter-State_Equalisation_of_Health_Expenditures_in_Indian_Union.pdf
49. Ravishankar V, Mishra D, Wes M, Harris C, Pritchett L, Menon V. India - Orissa in transition: challenges for 2006-2010. 2008.<http://www-wds.worldbank.org/external/default/WDSContentServer/WDSP/IB/2009/01/07/000333038_20090107233417/Rendered/PDF/446120ESW0P10210Box334115B01PUBLIC1.pdf>
50. Saggurti N, Verman R, Achyut P, Ramarao S, Jain A. Patterns and implication of male migration for HIV prevention strategies in Karnataka, India. 2008. https://www.popcouncil.org/pdfs/India_TechBriefKarnataka.pdf
51. Sakthivel S, Nagpal S. Tracking the flow of funds in the HIV/AIDS Sector in India - Using the National Health Accounts (NHA) Framework. 2008. http://www.whoindia.org/EN/Section2/Section232_1557.htm
52. Santhya K, Haberland N, Das A, Lakhani A, Ram F, Sinha R, Ram U, Mohanty S. Empowering married young women and improving their sexual and reproductive health: Effects of the first-time parents project. 2008. http://www.popcouncil.org/pdfs/India_FirstTimeParents.pdf
53. Santhya K, Jejeebhoy S, Ghosh S. Early marriage and sexual and reproductive health risks: Experience of young women and men in Andhra Pradesh and Madhya Pradesh, India. 2008. http://www.popcouncil.org/pdfs/India_EarlyMarriageSRH.pdf
54. Sharma S. Childhood mortality and health in India. 2008. http://www.iegindia.org/workpap/wp292.pdf
55. Sinha D. Empowering communities to make pregnancy safer: An intervention in rural Andhra Pradesh. 2008. http://www.popcouncil.org/pdfs/wp/India_HPIF/005.pdf
56. Sunley E. India: The tax treatment of bidis. 2008.<http://www.tobaccofreeunion.org/files/44.pdf>
57. United Nations Development Programme. Back to work: A rapid assessment of the impact of ARV therapy on PLHIV's work life. 2008. http://data.undp.org.in/hiv/iec/BTW%20Study.pdf
58. Varkey L, Mishra A, Khan M. Creating the conditions for scale-up of the men in maternity intervention in India. 2008.<http://pdf.usaid.gov/pdf_docs/PNADL823.pdf>
59. Verma R, Pulwerwitz J, Mahendra V, Khandekar S, Singh A, Das S, Mehra S, Nura A, Barker G. Promoting gender equity as a strategy to reduce HIV risk and gender-based violence among young men in India. 2008. http://www.popcouncil.org/pdfs/horizons/India_GenderNorms.pdf
60. Vora K, Mavalankar D, Ramani K, Upadhyaya M, Sharma B. Maternal health situation in India: A case study. 2008. http://www.iimahd.ernet.in/publications/data/2008-03-02Mavalankar.pdf
61. Zachariah K, Rajan S. Costs of basic services in Kerala 2007: Education, health, childbirth and finance (loans). 2008. http://cds.edu/download_files/wp406.pdf
62. Establishing principles on which to develop standards for good governance & professional management of public health sector at state & district levels. 2008. [http://94.76.210.161/index.php?option=com_docman&task=doc_download&gid=173&Itemid=115](http://www.nhsrcindia.org/index.php?option=com_docman&task=cat_view&gid=251&Itemid=108&limitstart=10)
